# Ferroptosis in oligodendrocyte progenitor cells mediates white matter injury after hemorrhagic stroke

**DOI:** 10.1038/s41419-022-04712-0

**Published:** 2022-03-23

**Authors:** Danmin Shen, Weihua Wu, Jing Liu, Ting Lan, Zhongnan Xiao, Kaiyuan Gai, Liye Hu, Zhaoli Luo, Chao Wei, Xiaotong Wang, Yabin Lu, Yamei Wang, Chenguang Zhang, Peipei Wang, Zhentao Zuo, Fei Yang, Qian Li

**Affiliations:** 1 Department of Biochemistry and Molecular Biology, School of Basic Medical Sciences, Capital Medical University, Beijing, 100069 China; 2 Department of Neurobiology, School of Basic Medical Sciences, Capital Medical University, Beijing, 100069 China; 3 School of Basic Medical Sciences, Capital Medical University, Beijing, 100069 China; 4 State Key Laboratory of Brain and Cognitive Science, Institute of Biophysics, Chinese Academy of Sciences, Beijing, 100101 China; 5 Hefei Comprehensive National Science Center, Institute of Artificial Intelligence, Hefei, China; 6 Sino-Danish College, University of Chinese Academy of Sciences, Beijing, 100049 China; 7 Advanced Innovation Center for Human Brain Protection, Beijing Key Laboratory of Neural Regeneration and Repair, Capital Medical University, Beijing, 100069 China

**Keywords:** Stroke, Cell death

## Abstract

Oligodendrocyte progenitor cells (OPCs) differentiate to myelin-producing mature oligodendrocytes and enwrap growing or demyelinated axons during development and post central nervous diseases. Failure of remyelination owing to cell death or undifferentiation of OPCs contributes to severe neurologic deficits and motor dysfunction. However, how to prevent the cell death of OPCs is still poorly understood, especially in hemorrhagic diseases. In the current study, we injected autologous blood into the mouse lateral ventricular to study the hemorrhage-induced OPC cell death in vivo. The integrity of the myelin sheath of the corpus callosum was disrupted post intraventricular hemorrhage (IVH) assessed by using magnetic resonance imaging, immunostaining, and transmission electron microscopy. Consistent with the severe demethylation, we observed massive cell death of oligodendrocyte lineages in the periventricular area. In addition, we found that ferroptosis is the major cell death form in Hemin-induced OPC death by using RNA-seq analysis, and the mechanism was glutathione peroxidase 4 activity reduction-resulted lipid peroxide accumulation. Furthermore, inhibition of ferroptosis rescued OPC cell death in vitro, and in vivo attenuated IVH-induced white matter injury and promoted recovery of neurological function. These data demonstrate that ferroptosis is an essential form of OPC cell death in hemorrhagic stroke, and rescuing ferroptotic OPCs could serve as a therapeutic target for stroke and related diseases.

## Introduction

Intracerebral hemorrhage (ICH) accounts for 10–30% of all stroke subtypes, but with mortality of 50% at 1 year [1, 2]. The most frequent occurrence location of ICH is basal ganglia and thalamus, which are rich in white matter fibers [3]. When ICH occurs, blood enters the brain parenchyma from the ruptured blood vessel, leading to primary and secondary brain injury, both of which impact the structure and function of white matter fibers [4, 5]. Studies have reported that white matter injury (WMI) often occurs after ICH, and is considered an important predictor of the outcome [6]. However, most of the previous treatments for ICH focus on neuronal protection but paid insufficient attention to the protection of WMI. Therefore, the repair of WMI is expected to become a new treatment strategy for ICH.

Oligodendrocyte progenitor cells (OPCs) present as a pool of migratory and proliferative progenitor cells in the adult central nervous system (CNS), which differentiate into mature oligodendrocytes and re-myelinate damaged axons in diseases such as multiple sclerosis and stroke [7]. However, OPCs and oligodendrocytes are both vulnerable to cytotoxic and excitotoxic factors, and in vivo, how to differentiate OPCs to mature oligodendrocytes and remyelinate damaged axons has always been a difficult problem to overcome [8, 9]. Therefore, inhibiting OPC cell death and maintaining the stability of the OPC pool is the prerequisite for myelin repair.

Ferroptosis is a newly identified type of cell death, driven by iron-dependent phospholipid peroxidation [10]. This unique cell death is regulated by multiple cellular metabolic events, including iron metabolism, redox homeostasis, amino acid metabolism, mitochondrial function, and numerous signaling pathways [11]. Glutathione peroxidase 4 (GPx4) is an antioxidant enzyme catalyzing the glutathione (GSH)-dependent reduction of membrane lipid hydroperoxides (L-OOH) to lipid alcohols (L-OH), which limits the lipid peroxide in the cell membrane. Cystine/glutamate antiporter system χ_c_^–^/GSH/GPX4 axis is a classical pathway protecting against ferroptosis, in which GPx4 is considered to be the key regulator in cancer cells and neurons [12–15]. Although we and others have demonstrated that ferroptosis is an important cell death form in cancers and CNS diseases, such as stroke [10, 14–17], whether ferroptosis contributes to cell death of OPCs and rescuing ferroptotic OPCs protects hemorrhagic brain are unclear.

In this study, we used the intraventricular hemorrhage (IVH) model to study OPC cell death post hemorrhage specifically. We found that IVH induced severe WMI and massive cell death of OPCs post-IVH. As an in vitro hemorrhagic model, Hemin induced accumulation of lipid reactive oxygen species (ROS) and subsequent ferroptosis. We additionally identified that downregulation of the expression of GPx4 was the major cause of ferroptosis in OPCs. Furthermore, administration with the ferroptosis inhibitor reduced OPC cell death in vitro, and improved WMI and related neurobehavior dysfunction in vivo. Together, these findings will advance the understanding of ferroptosis of OPCs and will be a viable treatment strategy for post-hemorrhagic injury.

## Results

### The IVH causes severe demyelination of white matter tracks in vivo

**Fig. 1:**
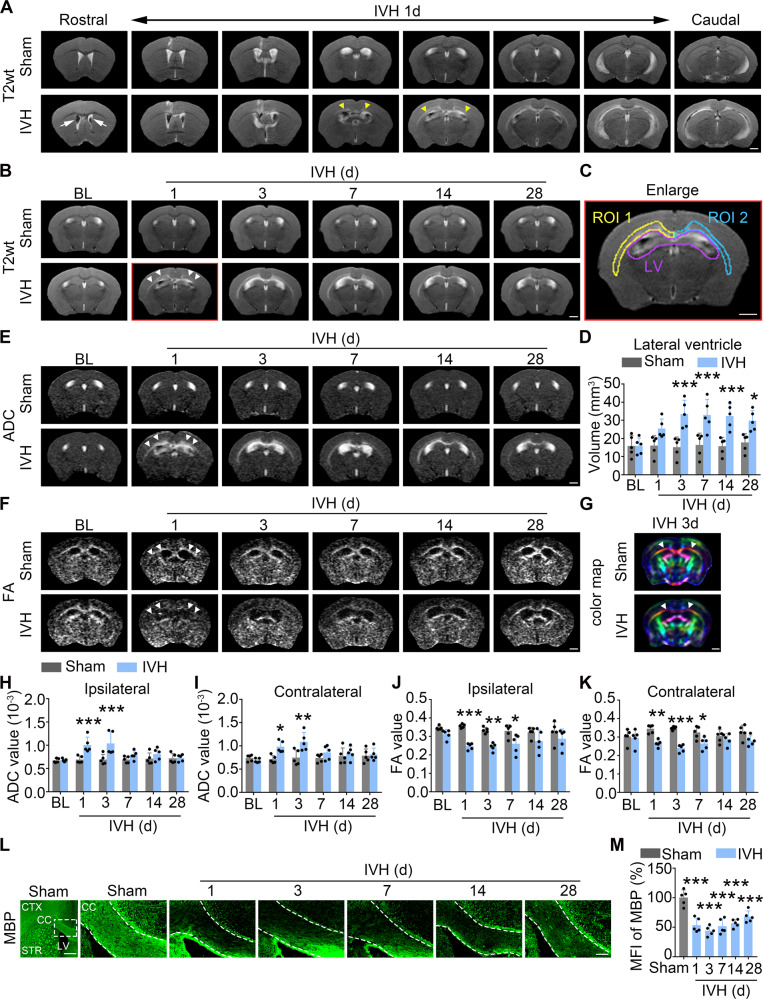
Sever demyelination in corpus callosum post-IVH. **A** Representative coronal views of T2wt MRI of sham and IVH mouse at 1d after surgery. **B** Longitudinal T2-weighted images show coronal views of IVH or sham mouse brain at different time points. **C** The ipsilateral and contralateral corpus callosum (CC) were set to region of interest (ROI) 1 and 2, respectively. **D** Quantification of the volume of bilateral lateral ventricles in IVH and sham mice. **E** Representative ADC map of sham and IVH mice at different time points after surgery. **F** Representative FA map of sham and IVH mice at different time points after surgery. **G** Color map of sham and IVH mice at 3d after surgery. Color codes to give diffusion tensor directions: red represents tracts running left to right; green is anterior to posterior; blue is superior to inferior. **H**, **I** Quantification of ADC values of ipsilateral and contralateral CC in IVH and sham mice. **J**, **K** Quantification of FA values of ipsilateral and contralateral CC in IVH and sham mice. **L**, **M** Myelin integrity was measured as the intensity of MBP immunostaining. Representative images (**L**) and quantifications (**M**) are shown. Data are calculated as fold change of sham. *CC* corpus callosum, *CTX* cortex, *LV* lateral ventricle. Results are presented as scatter plots (mean ± SD). **D**, **H**–**K** Two-way ANOVA with Sidak’s multiple-comparison test. **M** one-way ANOVA with Dunnett’s multiple-comparison test. *p < 0.05, **p < 0.01, ***p < 0.001 vs. corresponding sham. Each group contained five (**D**, **H**–**K**, **M**) animals. Experiments were repeated at least three times independently. Scale bar: (**A**–**G**) 1 mm, (**L**) left panel: 1 mm, right panels: 100 µm.

Hemorrhagic stroke causes both gray and white matter injuries [18], and magnetic resonance imaging (MRI) is a powerful tool to evaluate the severity of gray and white matter injuries in stroke [19]. T2-weighted (T2wt) MRI is used to define the area of the lesion, apparent diffusion coefficient (ADC) is used to evaluate the edema, and fractional anisotropy (FA) of diffusion tensor imaging (DTI) allows for quantitative evaluation of the structural integrity of white matter tracts [20]. To exclude the effects of massive neuronal death and to study the role of oligodendrocyte lineage cell death in the outcomes of hemorrhagic stroke, we injected autologous blood into the left ventricle of mice and performed MRI at different time points post-surgery. Lateral ventricular (LV) and corpus callosum (CC, the largest commissural fibers that link the cerebral cortex of both hemispheres in the brain) was manually delineated in T2wt MRI. T2wt MRI showed that the two lateral ventricles contained low signal intensities in the IVH group, indicating that IVH mice exhibited bilateral ventricular system hemorrhage (Fig. 1A). Additionally, gradually high signals from rostral to caudal in bilateral CC were observed (Fig. 1A). We quantified the volume of lateral ventricle, the sizes were increased from day 3, and maintained until day 28 (Fig. 1B, D), suggesting that IVH mice were complicated with post-hemorrhagic hydrocephalus.

Since the abnormal signal of bilateral CC was observed in IVH mice (Fig. 1B), we next set the CC as a region of interest (ROI) (Fig. 1C), and quantified the ADC and FA values of it in IVH and sham mice post-injury. Compared with sham mice, the ADC values were significantly increased on days 1 and 3 (Fig. 1E, H, I), indicating severe edema in the CC post-IVH. Changes in FA values in the bilateral CC showed the opposite trend, with FA values decreasing from day 1 to day 7, and reaching the lowest on day 3 (Fig. 1F, J, K). In addition, coronal, direction-coded color DTI images showed a thinner tract of bilateral CC (Fig. 1G), which indicates severe demyelination in the CC post-IVH.

We further confirmed the damage of white matter tracks by using immunostaining of myelin basic protein (MBP), the major constituent of the myelin sheath of oligodendrocytes. Paralleling with the changes observed with MRI, the mean fluorescence intensity of MBP in the CC was significantly reduced after IVH (Fig. 1L, M). These data suggest that disrupted white matter tracks in the bilateral side of mouse brains have not recovered until 4 weeks after IVH.

### Oligodendrocyte linage cell death and iron deposition in the peri-lateral ventricle area after IVH

**Fig. 2:**
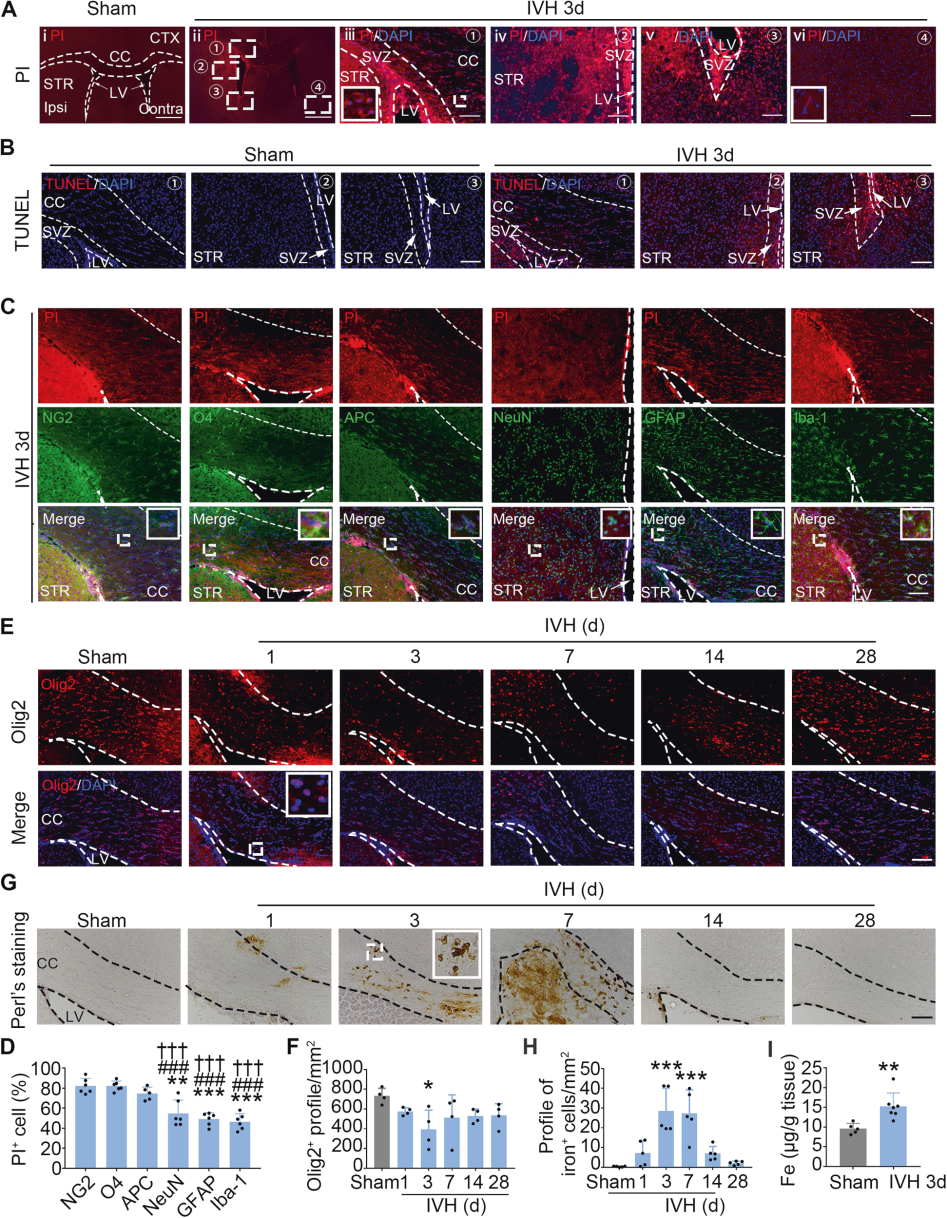
Oligodendrocyte linage cell death in peri-lateral ventricle area after IVH. **A** Representative views of the distribution of propidium iodide (PI)^+^ cells in peri-lateral ventricle area in sham (i) and IVH mice (ii–vi). Dashed squares (ii) indicate the enlarged areas of imaging in the periventricular area (iii–vi). DAPI stains the nuclei. **B** Brain sections were stained with TUNEL. Representative views of the peri-lateral ventricle area of sham (left) and IVH (right) mice are shown. **C**, **D** Immunostaining using indicated antibodies with PI on 3d after IVH. Representative images (**C**) and corresponding quantification of the ratio of double-positive cells among total cells labeled with each marker (**D**) in the peri-lateral ventricle area are shown. **E**, **F** Brain sections were stained with Olig2 and DAPI. Representative images (**E**) and quantification (**F**) of the profile of Olig2^+^ cells are shown. **G**, **H** Brain sections were stained with Perl’s stain. Representative images (**G**) and quantification (**H**) of the profile of iron^+^ cells are shown. **I** Fe content in the brain of sham and IVH was evaluated by ICP-MS. *CC* corpus callosum, *CTX* cortex, *LV* lateral ventricle, *SVZ* subventricular zone. Results are presented as scatter plots (mean ± SD). **D**, **F**, **H** One-way ANOVA with Tukey’s (**D**) or Dunnett’s (**F**, **H**) multiple-comparison test. **I** Two-tailed Student’s *t* test followed by Welch’s correction. *p < 0.05, **p < 0.01, ***p < 0.001 vs. NG2^+^ cell (**D**) or sham (**F**, **H**, **I**); ^###^*p* < 0.001 vs. O4^+^ cell; ^†††^*p* < 0.001 vs. APC^+^ cell. Each group contained four (**F**), five (**H**), five to six (**D**), or five to seven (**I**) animals. Experiments were repeated at least three times independently. Scale bars: **A**i, ii 800 μm; **A**iii–vi, **B**, **C**, **E**, **G** 100 μm.

We next evaluated the cell death of the major cell populations after IVH. Propidium iodide (PI) dye was injected into sham or IVH mice on day 3, and the profile of PI^+^ cells at the peri-bilateral ventricular areas was elevated (Fig. 2A). Compared with sham mouse brain (Fig. 2Ai), we found PI^+^ cells presented in the ipsilateral CC (Fig. 2Aiii), periventricular zone (Fig. 2Aiv), and ipsilateral subventricular (SVZ) zone (Fig. 2Av) of IVH mice, but not regions far from the lateral ventricle (Fig. 2Avi). We also obtained similar results by TUNEL staining (Fig. 2B). Meanwhile, we observed microglial activation at the periventricular zone in response to cell death in IVH animals (Fig. s1).

We then immunostained the ipsilateral periventricular brain region with markers of various cell populations and calculated the percentage of PI^+^ cells in each cell population. The proportions of co-localization of PI^+^ cells with NG2^+^ (OPCs), O4^+^ (pre-myelinating oligodendrocytes), and APC^+^ (myelin-producing mature oligodendrocytes) were higher. The rates of the PI^+^NeuN^+^ (neurons), PI^+^GFAP^+^ (astrocytes), and PI^+^Iba-1^+^ (microglia/macrophages) cells were relatively low (Fig. 2C, D). These data suggest that compared with other cell populations, oligodendrocyte lineage cells were especially vulnerable post-IVH. In order to explore the quantitative changes of oligodendrocyte linage cells after IVH, we quantified Olig2^+^ (a marker of oligodendrocyte linage) cells at different time points after IVH. The number of total Olig2 cells showed a trend toward decline in mice with IVH compared with sham, and significantly decreased on day 3 post-IVH (Fig. 2E, F).

Since iron toxicity is the major cause of cell death post-hemorrhagic stroke [17], we assessed the cellular iron accumulation using Perl’s staining. Abundant iron^+^ cells were found in the periventricular zone, and the number of iron^+^ cells was significantly increased on day 3 and day 7 after IVH (Fig. 2G, H). The increased content of iron ions in the IVH animals was further confirmed with inductively coupled plasma-mass spectrometry (ICP-MS) (Fig. 2I).

As the WMI (decreased FA value and MBP expression), oligodendrocyte lineage cell death, and iron overload timelines were completely matched, we speculated that the cell death of oligodendrocyte lineage was the major cause of WMI, and the overload of intracellular iron was related to cell death of oligodendrocyte lineage in mice with IVH.

### Hemin induces ferroptosis in OPCs in vitro

**Fig. 3:**
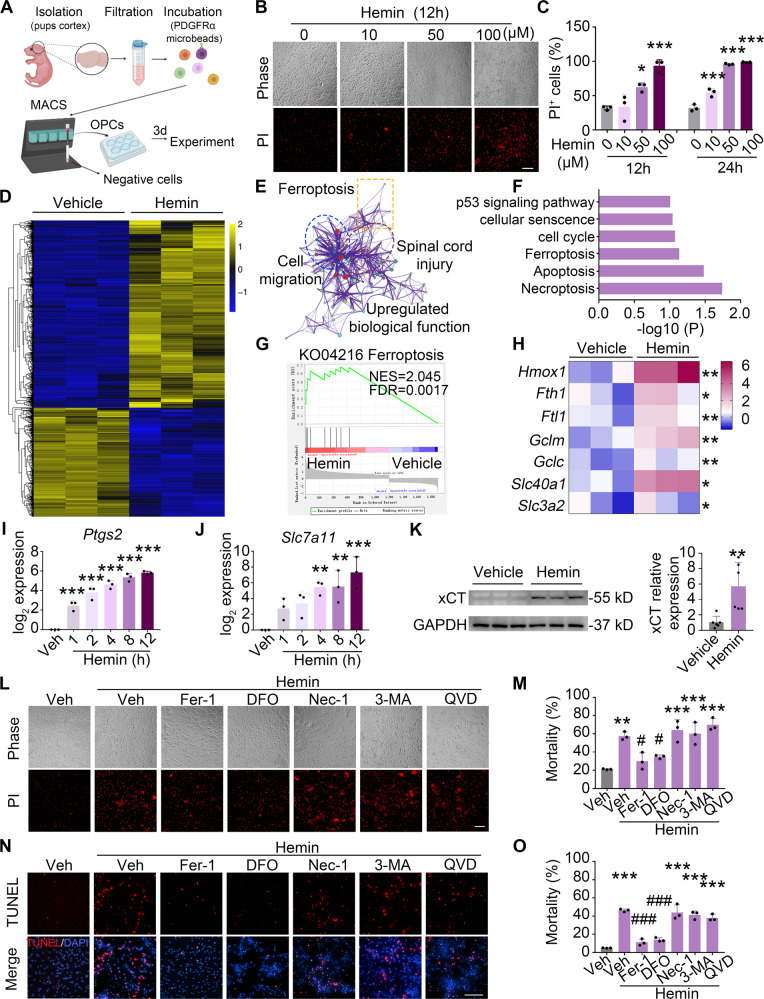
Hemin induces ferroptosis in OPCs in vitro. **A** Workflow of MACS sorting and primary culture of mouse OPCs. **B**, **C** OPCs were treated as indicated and PI staining was performed to assess cell death. Representative images (**B**) and quantifications (**C**) of PI^+^ cells are shown. **D**–**G** RNA-seq analyses on OPCs treated as indicated for 8 h. A heatmap of hierarchical clustering analysis of differentially expressed genes (**D**), a network plot of enriched GO biological processes (**E**), a KEGG pathways (**F**), and a GSEA analysis of ferroptosis (**G**) are shown. **H**–**J** OPCs were treated with Hemin as indicated and the mRNA level of ferroptosis-related genes was analyzed using RT-qPCR. *GAPDH* served as an internal control. **K** The expression of xCT protein level (encoded by *Slc7a11*) was detected by western blot. GAPDH served as an internal control. **L**, **M** OPCs were treated as indicated for 12 h. Representative images (**L**) and quantifications (**M**) of PI^+^ cells are shown. **N**, **O** OPCs were treated as indicated for 12 h. Representative images (**N**) and quantifications (**O**) of TUNEL^+^ cells are shown. *Fer-1* ferrostatin-1, *DFO* deferoxamine, *Nec-1* necrostatin-1, *3-MA* 3-methyladenine, *QVD* Q-VD-OPh. Results are presented as scatter plots (mean ± SD). **H**, **K** two-tailed Student’s *t* test followed by Welch’s correction. **C**, **I**, **J** One-way ANOVA with Dunnett’s multiple-comparison test; **M**, **O** one-way ANOVA with Tukey’s multiple-comparison test. *p < 0.05, **p < 0.01, ***p < 0.001 vs. corresponding vehicle, ^#^*p* < 0.05, ^###^*p* < 0.001 vs. Hemin+vehicle. Each experiment was repeated three times independently. Scale bars: **B**, **L**, **N** 100 μm.

To explore the cell death form of OPCs and its mechanism after IVH, we cultured mouse primary OPCs in vitro using magnetic-activated cell sorting (Fig. 3A). The purity of OPCs was determined using immunostaining (Fig. s2A), and the viability of OPCs was assessed with both PI and TUNEL staining (Fig. s2B). Hemin, a hemoglobin degradation product, is often used to mimic hemorrhagic damage in vitro [21] and was used in this study as an in vitro IVH model. PI staining showed that Hemin at a concentration range of 10–100 μM induced dose-dependent cell death of OPCs at 12 and 24 h (Fig. 3B, C). Then, RNA-seq was performed to further elucidate the mechanisms underlying the Hemin-induced cell death of OPCs. A total of 1902 genes showed differential expression in Hemin-treated OPCs compared with vehicle-treated OPCs (*p* < 0.05 and fold change > 2), among which 1202 genes were upregulated, and 700 genes were downregulated (Fig. 3D). The purity of OPC cultures was further confirmed by the value of fragments per kilobase of transcript per million mapped fragments (FPKM) of OPC markers obtained from RNA-seq data (Fig. s2C). The gene ontology (GO) enrichment was analyzed and the network plot revealed the relationships between the main regulated biological processes (*p* < 0.05 and fold change > 3), which were related to ferroptosis, spinal cord injury, and cell migration (Fig. 3E). The Kyoto Encyclopedia of Genes and Genomes (KEGG) pathway enrichment analysis based on the RNA-seq data showed six enriched pathways relating to cell growth and death, including the p53 signaling pathway, cellular senescence, cell cycle, ferroptosis, apoptosis, necroptosis (Fig. 3F).

Combining the above results, we focused on ferroptosis, a unique cell death form driven by iron-dependent phospholipid peroxidation, but rarely identified in OPCs [11]. By using Gene-Set Enrichment Analysis (GSEA), we noticed that regulation of the ferroptosis pathway was significantly enriched in Hemin-treated OPCs (Fig. 3G). We verified the expression of ferroptosis-related genes from the RNA-seq results by RT-qPCR, and they were significantly upregulated after Hemin treatment (Fig. 3H). In addition, we confirmed that Hemin led to a time-dependent upregulation of vital genes involved in ferroptosis, such as *Ptgs2* (the gene overexpressed in ferroptotic cells and serves as a marker of ferroptosis [12]) and *Slc7a11* (the gene encodes xCT, a subunit of system χ_c_^–^ importing cystine [10, 14]) (Fig. 3I, J). We further confirmed that the protein level of xCT was increased after Hemin treatment (Fig. 3K).

To determine that ferroptosis was the major cell death form in Hemin-induced cell death, OPCs were treated with Hemin or with various cell death inhibitors: ferroptosis (Fer-1 and DFO), necroptosis (Nec-1), autophagy (3-MA), and apoptosis (QVD). The results showed that only ferroptosis inhibitors, Fer-1 and DFO, rescued cell death of OPCs efficiently (Fig. 3L–O), suggesting that the major cell death form caused by Hemin was ferroptosis in OPCs.

### Hemin induces ferroptosis in OPCs by decreasing the expression of GPx4 in vitro

**Fig. 4:**
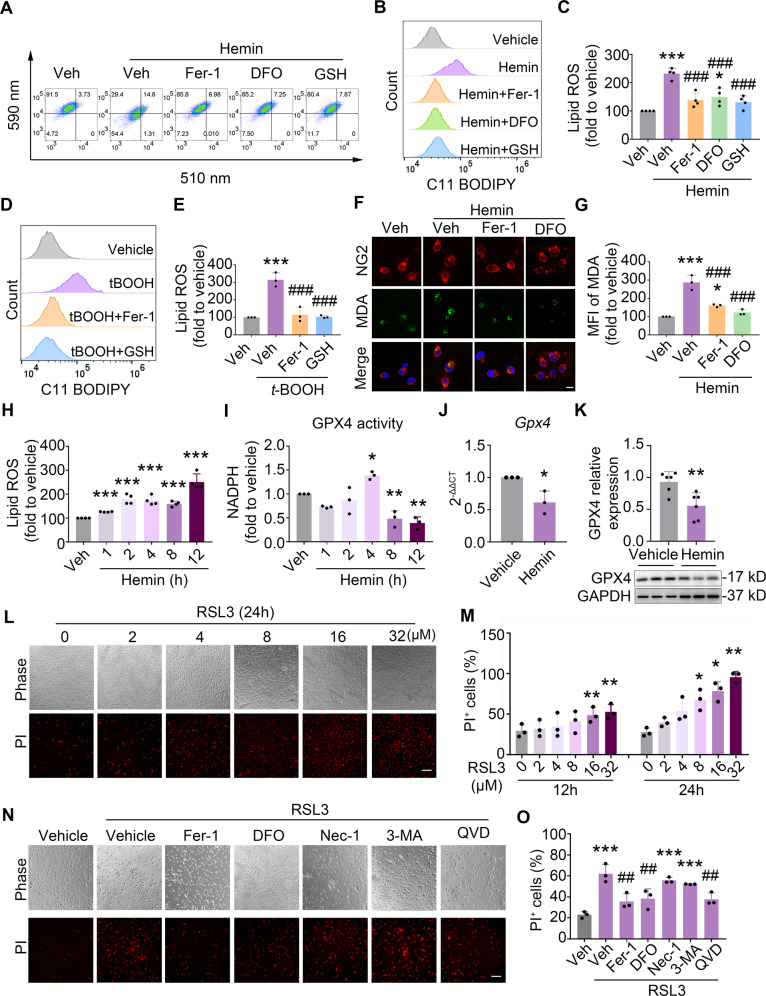
Hemin induces OPC ferroptosis by decreasing *GPx4* expression in vitro. **A**–**E** OPCs were treated as indicated and the accumulation of lipid ROS was assessed by C11 BODIPY staining followed by flow cytometry analysis. Representative images of double fluorescence dot plots (**A**), histogram (**B**, **D**), and quantifications (**C**, **E**) of flow cytometry are shown. **F**, **G** OPCs were treated as indicated and the lipid peroxidative product MDA content was assessed by immunostaining of NG2 and MDA. Representative images (**F**) and quantifications (**G**) are shown. DAPI stains nuclei. **H** OPCs were treated as indicated and lipid ROS accumulation was quantified by flow cytometry. **I** OPCs were treated as indicated and GPx4 activity was determined using a commercial kit. **J**, **K** OPCs were treated with Hemin for 12 h. The total mRNA and protein were extracted and the expression of *Gpx4* was assessed using RT-qPCR and western blot. GAPDH served as an internal control. **L**–**O** OPCs were treated as indicated. Representative images (**L**, **N**) and quantifications (**M**, **O**) of PI staining are shown. Results are presented as scatter plots (mean ± SD). **C**, **E**, **G**, **O** One-way ANOVA with Tukey’s multiple-comparison test; **H**, **I**, **M** one-way ANOVA with Dunnett’s multiple-comparison test; **J**, **K** two-tailed Student’s *t* test followed by Welch’s correction. *p < 0.05, **p < 0.01, ***p < 0.001 vs. corresponding vehicle, ^#^*p* < 0.05, ^##^*p* < 0.01, ^###^*p* < 0.001 vs. Hemin+Veh (**C**, **G**), *t*-BOOH + Veh (**E**) or RSL3 + Veh (**O**). Each experiment was repeated three (**E**, **G**, **I**, **J**, **M**, **O**), four (**C**, **H**), or six (**K**) times independently. Scale bars: (**F**) 25 μm; (**L**), (**N**) 100 μm.

Accumulation of lipid peroxidation is one of the hallmarks of ferroptosis [22]. We assessed lipid reactive oxygen species (ROS) accumulation by using C11 BODIPY 581/591 fluorescent probes. As glutathione (GSH) synthesis of antioxidants plays an indispensable role in preventing lipid peroxidation during ferroptosis [13], GSH was used as a positive antioxidant here. Hemin treatment increased the accumulation of lipid ROS, while GSH and ferroptosis inhibitors (Fer-1 and DFO) reduced the level of lipid ROS (Fig. 4A–C and s3). In addition, Tertiary-Butylhydroperoxide (*t*-BOOH) is a widely used inducer of oxidative stress and a scavenger of GSH [23]. Treating cells with *t*-BOOH increased the accumulation of lipid ROS, and Fer-1 and GSH inhibited it as expected (Fig. 4D, E, and s4). To confirm the lipid peroxidation in OPCs, we detected malondialdehyde (MDA) content, an end-product of lipid peroxidation. MDA content was markedly increased after Hemin treatment and was reversed by treating with Fer-1 or DFO (Fig. 4F, G).

We and others have identified that GPx4 is the most important endogenous defensive enzyme that inhibits lipid peroxidation in ferroptosis after hemorrhage [14, 15, 24]. To explore whether GPx4 played a vital role in OPC ferroptosis as well, we measured lipid ROS levels and GPx4 activity at different time points after Hemin treatment. Responding to the accumulation of lipid ROS, GPx4 activity peaked at 4 h, but then decreased rapidly (Fig. 4H, I). We speculated that it may be due to the change of gene expression of *GPx4* after Hemin treatment. Indeed, compared with the vehicle, Hemin administration effectively reduced *Gpx4* expression in both levels of mRNA (Fig. 4J) and protein (Fig. 4K).

To investigate whether loss of GPx4 expression and activity was the main mechanism of OPC ferroptosis, an irreversible GPx4 inhibitor by covalently modifying its active-site Selenocysteine residue [13], RSL3, was incubated with OPCs. As expected, RSL3 induced time- and dose-dependent cell death of OPCs (Fig. 4L, M). Similarly, ferroptosis inhibitors, Fer-1 and DFO, rescued cell death induced by RSL3 (Fig. 4N, O). Taken together, these results indicate that the inactivation of GPx4 contributes to ferroptosis induced by Hemin in OPCs.

### Inhibition of ferroptosis rescues CC demyelination and improves long-term neurological functions after IVH in vivo

**Fig. 5:**
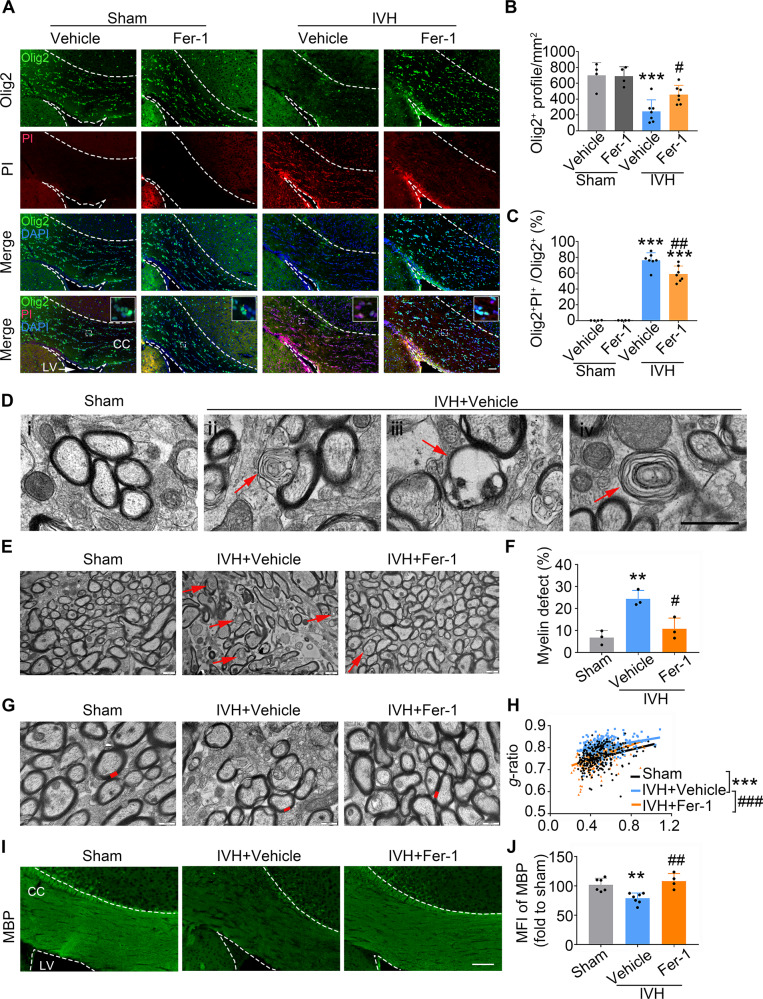
Ferroptosis inhibitor Fer-1 rescues corpus callosum demyelination after IVH in vivo. Mice received an injection of Fer-1 (i.p.) or vehicle 2 h after IVH and at daily intervals on days 1–7 d after surgery. **A**–**C** Immunostaining of Olig2 antibodies with PI on 3d after IVH. Representative images (**A**) and corresponding quantification of the Olig2^+^ cells (**B**) and the ratio of double-positive cells among total Olig2^+^ cells (**C**) in the peri-lateral ventricle area are shown. **D**–**H** Ultrastructural analyses of myelin integrity in the corpus callosum (CC) of indicated groups at 28 d after surgery using transmission electron microscopy. Representative TEM images of normal (**D**i) in sham and defected myelin sheath (**D**ii–iv) in IVH mouse brain. (**D**ii) defective myelin layers split and bulged to form a “myelin balloon”; (**D**iii) the myelin sheath around the axon was discontinuous and ruptured, and the axon within the myelin sheath has completely degenerated; (**D**iv) an axon was loosely wrapped by an abnormal myelin sheath. Representative TEM images (**E**) and quantifications (**F**) of defective myelin sheath in indicated groups are shown. Red arrows indicate defective myelin sheaths. Representative TEM images of myelin thickness (**G**, shown as red) and the quantification (**H**) of *g*-ratio of myelinated axons in the CC after IVH. (**I**, **J**) Brain sections were stained for MBP. Representative images (**I**) and quantifications (**J**) of mean fluorescence intensity of ipsilateral CC are shown. *CC* corpus callosum, *CTX* cortex, *LV* lateral ventricle. Results are presented as scatter plots (mean ± SD). **B**, **C**, **F**, **H**, **J** One-way ANOVA with Tukey’s multiple-comparison test. **p < 0.01, ***p < 0.001 vs. corresponding sham; ^#^*p* < 0.05, ^##^*p* < 0.01, ^###^*p* < 0.001 vs. IVH + vehicle. Each group contained three (**F**, **H**) or four–seven (**B**, **C**, **J**) animals. Axons calculated for evaluating *g*-ratio: sham *n* = 271, IVH + vehicle *n* = 280, IVH + Fer-1 *n* = 254. Experiments were repeated at least three times independently. Scale bars: **A**, **I** 100 μm; **D**, **E** 1 μm; **G** 500 nm.

Since Hemin induced ferroptosis in OPCs in vitro, we asked whether inhibiting ferroptosis would rescue WMI and improve neurobehavioral deficits after IVH in vivo. To this end, we injected IVH mice with ferroptosis inhibitor Fer-1 (*i.p*.). First, immunostaining showed that Fer-1 partially saved the decline in the total number of Olig2^+^ cells and prevented IVH-induced oligodendrocyte cell death on 3 days post-IVH (Fig. 5A–C). Next, transmission electron microscopy (TEM) evaluated ultrastructural myelin integrity and axonal myelination among groups on 28 days after surgery. Normal axons were tightly wrapped by dense myelin sheath in sham mouse brains (Fig. 5Di), whereas myelin sheath defects were observed in IVH mouse brains (Fig. 5Dii–iv).

To determine whether Fer-1 can improve myelin integrity and axonal myelination after IVH, myelin defect and thickness of myelin sheath were measured. The percentage of defect myelin increased significantly after IVH, and the integrity was improved by Fer-1 administration (Fig. 5E, F). The ratio of the inner axonal diameter to the total outer diameter of the myelinated fiber is called g-ratio and is used to assess the myelin thickness [25]. Results showed that the myelin sheath of IVH mice was significantly thinner, and Fer-1 treatment significantly reduced the g-ratio of axonal fibers (Fig. 5G, H). In addition, we immunostained brain sections with MBP, and administration of Fer-1 markedly increased MBP expression after IVH (Fig. 5I, J).

**Fig. 6:**
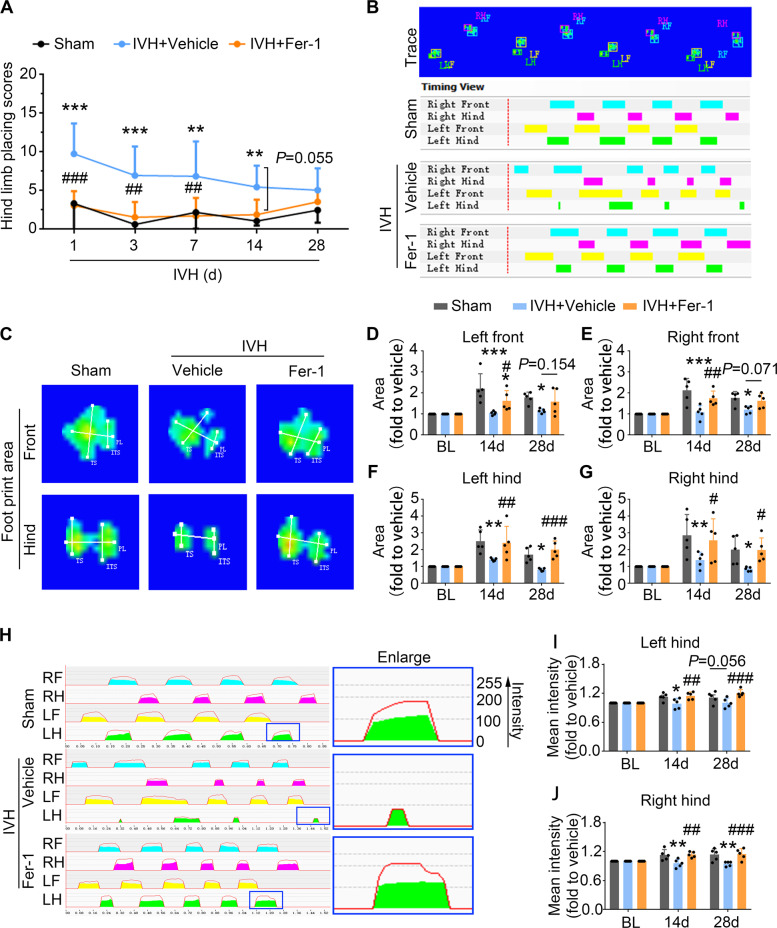
Administration of Fer-1 improves long-term neurological functions after IVH in vivo. Mice received injection of Fer-1 (i.p.) or vehicle 2 h after IVH and at daily intervals on days 1–7 d after surgery. **A** Hindlimb placement test was assessed at indicated time points. **B**–**J** The gait of mice was tested using the CatWalk XT system at indicated time points. **B** Representative footprints (upper panel) and print pattern (lower panel) are shown. Representative images (**C**) and quantifications (**D**–**G**) of the footprint area of four paws are shown. Representative images (**H**) and quantifications (**I**, **J**) of the mean intensity of hind limbs are shown. *RF* right front paw, *LF* left front paw, *RH* right hind paw, *LH* left hind paw. Results are presented as line graphs or scatter plots (mean ± SD). **A**, **D**–**G**, **I**, **J** Two-way ANOVA with Tukey’s multiple-comparison test. *p < 0.05, **p < 0.01, ***p < 0.001 vs. corresponding sham, ^#^*p* < 0.05, ^##^*p* < 0.01, ^###^*p* < 0.001 vs. corresponding IVH + vehicle. Each group contained six–nine (**A**), five (**D**–**G**, **I**, **J**) animals. Experiments were repeated at least three times independently.

Consistent with the role of Fer-1 in rescuing ferroptosis in OPCs and preserving myelin sheath, the hindlimb placing test scores were significantly reduced on 1, 3, 7, and 14 days in vehicle-treated IVH mice, whereas administration of Fer-1 significantly improved the motor function in corresponding time points (Fig. 6A). To further evaluate the neurobehavior in the long-term stage of injury unbiasedly, we performed gait analysis on 14 and 28 days after surgery with the CatWalk system, an automated gait analysis system, to analyze the walking pattern via a recording of footprints [26]. Differences were found in the walk pattern and footprint area of all limbs (Fig. 6B–G). Linked to these results, a significant difference was also observed in the intensity of bilateral hind limbs (Fig. 6H–J). All these parameters showed lower values for the ipsilateral and contralateral limbs of IVH in comparison to the sham mice, and were improved by Fer-1 treatment (Fig. 6B–J).

## Discussion

Oligodendrocytes are the myelinating cells in the central nervous system (CNS). They participate in myelin formation, accurate wrapping, and repair in development and various diseases [8]. Mature oligodendrocytes are derived from oligodendrocyte progenitor cells (OPCs), which originate from neuroepithelial progenitor cells of neuroepithelium in the embryonic neural tube and the periventricular germinal area (retain in adult animals and known as the subventricular zone, SVZ) during embryogenesis and early postnatal period [27]. The previous research has proved that OPCs originate in the SVZ and migrated into corpus callosum (CC), striatum, and fimbria fornix to differentiate into mature oligodendrocytes in adult mice [28], indicated that OPCs in the SVZ of the brain contribute to oligodendrogenesis throughout life and rescuing OPC cell death could be an efficient strategy to treat myelin defects-related CNS diseases.

In this study, we injected autologous blood into the lateral ventricle of mice to study the pathological process of hemorrhage on OPCs and to prevent the interference of neuronal ferroptosis in outcome elevations in vivo. After intraventricular stroke (IVH), blood enters the lateral ventricle, which first produces huge pressure and space-occupying effect on the structures around the ventricle, increases intracranial pressure, reduces blood flow and oxygen metabolism, and may destroy the permeability of the blood–brain barrier and blood-cerebrospinal fluid barrier [29]. Secondly, blood components, such as hemoglobin, heme, iron, and thrombin, will destroy periventricular structures and lead to cell death [30]. The blood-induced reactions, including oxidative stress, glutamate excitotoxicity, inflammation, deranged signaling pathways, and alteration of the extracellular matrix, all contribute to white matter injury (WMI) after hemorrhage [31]. During the acute stage after hemorrhage, various adverse factors lead to cell damage and even death of oligodendrocyte line, which induced inefficient production of new myelin sheaths. Therefore, protecting and rescuing damaged oligodendrocyte lineage cells is the basis and premise of remyelination. Our study focused on the inhibition of pathological processes by rescuing cell death of OPCs.

We observed that IVH significantly increased oligodendrocyte lineage cell death, especially NG2^+^ cells, which are progenitor and immature oligodendrocytes in periventricular areas. This is consistent with previous reports that among cells of oligodendrocyte lineage, APC^+^ mature oligodendrocytes are less vulnerable to injury compared with OPCs and immature oligodendrocytes [32]. Combined with recent research that hemoglobin causes damage to mitochondrial function in primary cultured OPCs and inhibits OPC proliferation [33], our work further emphasizes the importance of protecting OPC against cell death after hemorrhagic stroke to reduce WMI.

On the other hand, we observed a small number of other cells colocalized with PI, suggesting that cell death in OPCs is an important but not exclusive mechanism for WMI after IVH. Although a mechanistic contribution of OPC-specific cell death to WMI is reported here, it is conceivable that the crosstalk of OPCs and other cells, such as microglia/macrophages, astrocytes, and neurons, in the pathogenesis of WMI is also of significant importance and requires further studies.

Ferroptosis, a new form of iron‐dependent programmed cell death, has been shown to be involved in a range of CNS diseases, such as stroke, traumatic brain injury, neural degeneration, and autoimmune demyelination in the context of multiple sclerosis and its mouse model of EAE [14, 34–38]. Previous studies on ferroptosis in the CNS mostly focused on neurons [39, 40] until a recent study using human and mouse Pelizaeus-Merzbacher disease (PMD) oligodendrocytes, demonstrated that *PLP1* gene mutation caused severe iron sensitivity and the cell death involved various cell pathogenetic mechanisms of iron-induced ferroptosis, ER stress and apoptosis, and these pathways in some PMD lines are alleviated by iron chelation [41]. It suggests that ferroptosis of OPCs plays an important role in the disease model caused by a special gene mutation. However, there are barely any studies on the involvement of OPC ferroptosis in WMI after hemorrhage stroke.

Here, our report demonstrated that ferroptosis inhibition reduced cell death of OPCs and promoted myelination in a mouse model of hemorrhagic stroke. Lipid peroxidation has been recognized as a fundamental part of ferroptosis execution [42] and the overexpression *Ptgs2* is a recognized marker of ferroptosis [13, 14]. In our study, primary OPC cultures under the administration of Hemin have shown an increase in lipid peroxidation and the expression of *Ptgs2*, and inhibition of ferroptosis with Fer-1 has offered cytoprotection. Combined with RNA-seq analysis, suggesting ferroptosis is a major mechanism of OPC cell death. In terms of mechanism, cystine/glutathione (GSH)/glutathione peroxidase 4 (GPx4) is the canonical pathway in ferroptosis prevention, in which GPx4 is considered as the central regulator [13, 43].

GPx4, a member of selenoenzyme, specifically reduces lipid peroxidation by utilizing GSH [44]. It is a major hydroperoxide scavenger, and plays a vital role in the suppression of ferroptosis [24]. To study the mechanisms underlying Hemin administration, we performed RT-qPCR and Western blot and found the expression of *Gpx4* significantly decreased by Hemin in OPCs, as well as the GPx activity. However, the expression of *Slc7a11* increased protectively. In addition, GPx4 specific inhibitor RSL3 resulted in time - and dose-dependent ferroptosis of OPCs, which was successfully rescued by ferroptosis inhibitors, further confirming that Hemin causes ferroptosis in OPCs by affecting GPx4. Unexpectedly, pan-caspase inhibitor, Q-VD-OPh (QVD), also rescued cell death induced by RSL3 (pan-caspase inhibitors cannot inhibit RSL3-induced cell death in neurons and cancer cells [45, 46]). We suspect that QVD inhibited caspase-11 activation and subsequent Gasdermin D cleavage in RSL3-induced OPCs as reported in myeloid lineage cells [47].

In conclusion, our results demonstrated that ferroptosis occurred in Hemin-induced OPCs in a GPx4-dependent manner, and ferroptotic OPCs contributed greatly in WMI post-IVH. Ferroptosis inhibitors, such as Fer-1, alleviated the OPC ferroptosis in vitro and WMI-related neurobehavioral deficits in vivo, showing the important role of ferroptosis in OPC cell death. Targeting OPC ferroptosis is a promising therapeutic strategy for hemorrhagic stroke and WMI-related diseases in clinics.

## Methods

### Experimental design

Three or more independent experiments were performed for all experiments. Animals and cell cultures for each group were randomized with the website www.randomization.com. Treatment, data collection, and data analyses were blinded by using different investigators or by masking sample labels.

For in vitro experiments, we tested different dosages of Hemin (0, 10, 50, or 100 μM) and RSL3 (0, 2, 4, 8, 16, or 32 μM) at 12 and 24 h respectively. We added 2 μM Ferrostatin-1 (Fer-1, S7243, Selleck), 100 μM Deferoxamine (DFO, Y0001937, Sigma), 100 μM Necrostatin-1 (Nec-1, N9037, Sigma), 1 mM 3-Methyladenine (3-MA, HY-19312, MCE, USA), or 10 μM Q-VD-OPh (SML0063, Sigma) at the same time with Hemin or RSL3 based on previously published work [21, 48]. We treated cells with 20 μM *t*-BOOH (458139, Sigma, USA) for 12 h, with or without Fer-1 or 0.5 mM GSH (G4251, Sigma, USA) at the same time. Cells were collected for PI staining, TUNEL staining, immunofluorescence staining, flow cytometry, GPx4 activity assay, RT-qPCR, and RNA-seq.

For in vivo experiments, Fer-1 (2 mg/kg) or vehicle (0.1% DMSO (D1418, Sigma), 2.5% PEG300 (202371, Sigma) and 0.25% Tween80 (P1754, Sigma) in saline) was injected daily (*i.p*.) for the initial 7 days beginning 2 h after IVH. The assessment included behavior tests, neuroimaging, histology, and TEM.

### Animals

All animal studies were conducted in accordance with National Institutes of Health guidelines and were approved by the Capital Medical University Animal Care and Use Committee. C57BL/6 male mice (6–8 weeks old, 18–24 g) and neonatal mouse pups (P0-2, no limitation with sex) were obtained from Charles River Laboratories and maintained in the Capital Medical University animal facility.

### IVH mouse model

The intracerebroventricular injection was performed as previously described [49, 50]. Briefly, the mice were anesthetized with 1–2.5% isoflurane (R510-22, RWD, Shenzhen, China) and were placed in a stereotaxic apparatus (E04545, RWD). After adjusting the bregma and the rear fontanel to the same horizontal position, set the x, y, and z coordinates to zero. Autologous blood taken from the tail (25 µL) was collected in a sterile syringe (79017, RWD). The needle was introduced into the left periventricular region of the mouse at the following coordinates relative to bregma: 0.8 mm lateral, 0.5 mm posterior, and 2.5 mm in depth. Blood was injected slowly at 5 µL/min, and the needle was kept in place for 10 min and then removed slowly to prevent the reflux of blood. The craniotomy was sealed with Super Glue (1469SB, 3 M, MN, USA). Sham-operated control mice received only needle insertion.

### MRI

Animals were anesthetized with 1–2% isoflurane in a mix of oxygen and air and placed in the animal holder. The mice were maintained at ~50 breaths per minute. We performed MRI experiment by Brucker Biospin 11.7 T MR scanner. As described previously [20], T2wt images were collected with the following parameters: echo time = 44 ms, repetition time = 3899 ms, echo spacing = 14.667 ms, RARE factor = 8, signal averages were 4, the field of view was 15 × 15 mm, slice thickness = 0.5 mm. DTI was performed by using a three-segments diffusion-weighted echo-planer imaging sequence with the following parameters: echo time = 19 ms, repetition time = 1500 ms, averages = 1, *b* value = 1500 s/mm^2^, and the same field, thickness as the T2-wt images. The ROI was drawn manually in a blinded manner encompassing the EC and IC in the ipsilateral and contralateral hemispheres with MIPAV software, and FA value and ADC value of ROI were measured by MATLAB software.

### Immunofluorescence staining

For in vivo experiment, mice were anesthetized with 2–3% isoflurane and then cardiac perfused with 0.01 M PBS (4 °C), followed by 4% paraformaldehyde (PFA, P804536, MACKLIN, Shanghai, China). The brains were post-fixed in 4% PFA overnight at 4 °C and then dehydrated in 20% and 30% sucrose solutions (10021418, SCR, Shanghai, China) for 72 h at 4 °C successively. The brain tissues were then embedded with OCT and stored at −80 °C. Frozen tissues were cut into consecutive coronal sections (16 μm). slides were washed with 0.01 M PBS (P1010, Solarbio, Beijing, China) three times for 5 min then developed an antigen-retrieval protocol using a high temperature 0.01 M citric acid buffer (pH 6.0) (10007118, SCR) for 10 min. The sections were incubated in 1% Triton X-100 (T9284, MO, Sigma) in PBS for 15 min at room temperature followed by PBS three times. And then the sections were incubated in 0.3% H_2_O_2_ (10011208, SCR) for 10 min followed by PBS. After being blocked with 5% goat serum (C0265, Beyotime, Shanghai, China) in 0.3% Triton X-100 for 1 h at room temperature, the sections were incubated at 4 °C overnight with primary antibody including: anti-Olig2 (1:300, ab9610, Millipore, MO, USA), anti-NG2 (1:500, ab5320, Millipore), anti-O4 (1:200, O7139, Sigma), anti-APC (1:500, OP80, Millipore), anti-Iba-1 (1:500, 019-19741, Wako, Kanagawa, Japan), anti-GFAP (1:1000, G3893, Sigma), anti-NeuN (1:500, D3S3I, CST, MA, USA), anti-MBP (1:200, sc271524, Santa Cruz, CA, USA). The sections were washed with PBS and incubated with fluorescence-conjugated secondary antibodies (1:1000, Alexa Fluor 594 goat-anti-rabbit IgG, A-11012, Alexa Fluor 594 goat-anti-mouse IgG, A-11005, Alexa Fluor 488 goat-anti-rabbit IgG, A-11008 and Alexa Fluor 488 goat-anti-mouse IgG, A-11001, all from Thermo Fisher Scientific, MA, USA) for 1 h at room temperature. After washing with PBS, sections were mounted in VECTASHIELD mounting medium with DAPI (H-1200, VECTASHIELD, CA, USA).

For in vitro experiment, cells were fixed in 4% PFA solution for 15 min at room temperature followed by PBS three times. Then, cells were incubated with 5% goat serum for 1 h before being incubated with the primary antibodies of anti-NG2 and anti-MDA (ab243066, 1:500, Abcam, Cambridge, UK) overnight at 4°C. Incubation was continued with fluorescence-conjugated secondary antibodies for 1 h. Nuclei were stained with 1 μg/ml 4’,6-diamidino-2-phenylindole (DAPI, 4083 S, CST) for 10 min.

Images were acquired by fluorescence microscope (Nikon ECLIPSE Ci) or confocal microscope (Leica TCS SP8 STED) and evaluated double-blinded by the Image J software (NIH, USA).

### PI staining

Cell death was determined by PI staining. For in vivo experiments, PI was introduced via intracerebroventricular injection as described previously [51]. In brief, PI (1 mg/mL in 0.5 μL normal saline, P4170, Sigma) was given to mice (i.c.v.) at 3 days after surgery. mice were transcardially perfused with PBS and 4% PFA 20 min after PI injection. Brains were kept in 30% sucrose followed by frozen section.

For in vitro experiments, cells were incubated with 100 ng/μL PI for 30 min, and pictures were taken under a fluorescence microscope (Nikon ECLIPSE Ti). The percentage of cell death was calculated as the number of PI^+^ cells divided by the total number of cells.

### TdT-mediated dUTP nick-end labeling (TUNEL) staining

Cell death was determined by TUNEL staining with a one-step TUNEL apoptosis assay kit (C1090, Beyotime, China). In brief, cells or brain sections were fixed in 4% PFA and rinsed with PBS, and then permeabilized by 0.3% Triton X-100. Then cells or brain sections were incubated with a TUNEL reaction mixture of 30 °C for 1 h. Finally, the cells were incubated with DAPI (1 μg/ml, 4083 S, CST) in a humidified dark chamber. Cells were observed under a fluorescence microscope (Nikon ECLIPSE Ti), and the percentage of cell death was calculated as the number of TUNEL^+^ cells divided by the DAPI^+^ cells.

### Perl’s staining

Cellular iron accumulation was detected by Perl’s staining as described previously [17]. Sections were immersed in PBS for 5 min at four times and then incubated in a solution containing 20% HCl (7647-01-0, SCRC, Beijing, China) and 10% Potassium Ferrocyanide (P3289, Sigma) for 30 min with a gentle shake, followed by four washes in PBS. Then sections were incubated in 3,3’-diaminobenzidine (DAB) with the DAB staining kit (ZLI9017, SGB-BIO, Beijing, China) according to the manufacturer’s protocol. After washing 5 min in ddH2O, sections were incubated in hematoxylin staining solution (AR0713, DGCS, Beijing, China) for counterstaining and check with staining under the microscope. Rinse in distilled water five times and then dehydration with different concentrations of ethanol (10009218, SCRC) and xylenes (10023418, SCRC). Finally, neutral gum was used for mounting. Images around bilateral lateral ventricles were acquired by microscope (Nikon ECLIPSE) and cell counting was performed double-blinded by the Image J software.

### Mouse primary OPC cultures

Single cells were prepared from neonatal mouse (P0-2) cortices as described previously [52]. Primary OPCs were obtained from C57BL/6 pups using magnetic-activated cell sorting with the CD140 (PDGFRα) MicroBead Kit (130-101-502, Meltenyi, CA, USA) according to the manufacturer’s protocol. Primary OPCs were plated into poly-D,l-ornithine–coated (P0421, Sigma) culture plates, and expanded in growth medium:DMEM-F12 (C11330500BT, Gibco, USA) with 2% B27 (17504044, Invitrogen, USA), 20 ng/mL PDGF-AA (100-13 A, PeproTech, NJ, USA), 20 ng/mL bFGF (100-18B, PeproTech), 1% penicillin-streptomycin (30-002-CI, Corning, USA) for 3 days before experiments.

### Real-time quantitative polymerase chain reaction (RT-qPCR) and RNA-sequencing analysis

After drug treatment, mRNA of cells was extracted using TRIzol reagent (15596018, Invitrogen), and then total RNA was reverse transcribed into cDNA using Reverse Transcriptase kit (R323–01, Vazyme). Real-time PCR was performed by a 7500 Fast Real-Time PCR System (Applied Biosystems 7500) with the PowerUpTM SYBRTM Green Master Mix (A25742, Applied Biosystems). Each sample condition contains at least three biological replicates and all measurements were performed with technical replicates. Gene expression was normalized with *GAPDH* (Forward 5′-TGGATTTGGACGCATTGGTC-3′, Reverse 5′-TTTGCACTGGTACGTGTTGAT-3′). Primers used in this study are *Hmox1* (Forward 5′-AAGCCGAGAATGCTGAGTTCA-3′, Reverse 5′-GCCGTGTAGATATGGTACAAGGA-3′), *Fth1* (Forward 5′-CAAGTGCGCCAGAACTACCA-3′, Reverse 5′-GCCACATCATCTCGGTCAAAA-3′), *Ftl1* (Forward 5′-CCATCTGACCAACCTCCGC-3′, Reverse 5′-CGCTCAAAGAGATACTCGCC-3′), *Gclm* (Forward 5′-TGGAGCAGCTGTATCAGTGG-3′, Reverse 5′-AGAGCAGTTCTTTCGGGTCA-3′), *Gclc* (Forward 5′-GGGGTGACGAGGTGGAGTA-3′, Reverse 5′-GTTGGGGTTTGTCCTCTCCC-3′), *Slc40a1* (Forward 5′-TGTCAGCCTGCTGTTTGCAGGA-3′, Reverse 5′-TCTTGCAGCAACTGTGTCACCG) *Slc3a2* (Forward 5′-TGATGAATGCACCCTTGTACTTG-3′, Reverse GCTCCCCAGTGAAAGTGGA), *Slc7a11* (Forward 5′-GCTCGTAATACGCCCTGGAG-3′, Reverse 5′-GGAAAATCTGGATCCGGGCA-3′), *Ptgs2* (Forward 5′-TGAGCAACTATTCCAAACCAGC-3′, Reverse 5′-GCACGTAGTCTTCGATCACTATC-3′), and *GPx4* (Forward 5′-CTGCTCTTCCAGAGGTCCTG-3′, Reverse 5′-GAGGTGTCCACCAGAGAAGC-3′).

RNA-Seq analysis was performed using the Illumina HiSeq platform with vehicle and Hemin treatment to identify the differential expression genes. Each group had three samples. Briefly, total RNA was extracted from each sample, and the mRNAs were enriched with Oligo-d(T) beads and fragmented. Then, the cDNA was synthesized, and finally, the cDNA was amplified and purified. After the construction of the library, the insert fragment range of the library was detected by Agilent 2100 Bioanalyzer and the concentration of the library was quantified by ABI StepOnePlus Real-Time PCR system. After passing the quality inspection, the libraries were sequenced by Illumina HiSeq sequencer (PE 150). FASTQ reads were trimmed and required a minimum length of 20 bp. After filtering, the clean data reached an average of 6 G clean base per RNA sample. Next, we aligned clean reads to the reference genomeusing Hierarchical Indexing for Spliced Alignment of Transcripts (HISAT) [53], then quantified the gene expression and detected differential expression genes. Based on the comparison results, a new transcript prediction, differential splicing gene detection, SNP and indel detection, fusion gene detection. The known genes and new genes were analyzed quantitatively. A gene was considered to be differentially regulated when *P* < 0.05 and log2FCå 1. The screened differentially expressed genes were analyzed by GO function analysis, KEGG pathway function analysis, cluster analysis, protein-protein interaction network (https://metascape.org/), GSEA (https://www.gsea-msigdb.org/gsea/index.jsp).

### Western blot

Cultured cells were lysed with RIPA lysis buffer (AR0101, Boster, China) and a 10% protease inhibitor cocktail (04693132001, Roche, USA). The protein concentration was determined using the BCA protein assay kit (23221, Thermo, USA). The samples were electrophoresed on sodium dodecyl sulfate-polyacrylamide gel and transferred onto polyvinylidene difluoride membrane (1620177, Bio-rad, USA). Membranes were blocked with 5% non-fat milk and incubated with corresponding primary antibodies and horseradish peroxidase-conjugated secondary antibodies. Primary antibodies as follows: xCT (1:1000, ab37185, Abcam), GPX4 (1:1000, ab125066, Abcam), GAPDH (1:2000, 5174S, CST). Immunoreactive bands were visualized using chemiluminescent HRP substrate (90719, Millipore, USA) and analyzed using the Fusion system (Fusion fx 7 Spectra, Vilber, France). Results are expressed as a percentage of the values for GAPDH. All the original blots can be found in the supplementary files.

### Lipid peroxidation measurement

Lipid ROS was analyzed by BODIPY™ 581/591 C11 reagent manufacturer’s protocol (D3861, Invitrogen) with flow cytometry as described previously [22]. Approximately 2 × 10^5^ cells/well were seeded in 12-well plates 3 days followed by treatment with inhibitors or activators for 4 h. The cells were incubated with 10 μM Lipid Peroxidation Senser (BODIPY 581/591 C11 reagent) in a growth medium in the dark at 37 °C for 30 min. Remove media and wash cells three times with warmed PBS. The cellular fluorescence was analyzed by flow cytometry (LSR Fortessa SORP, BD) using PI and fluorescein isothiocyanate channels. The shift of the fluorescence emission peak from 590 nm to 510 nm gives the read-out for lipid peroxidation in cells. The data were analyzed by BD FACSDiva and FlowJo v10.

### GPx4 activity assay

Total GPx activity was measured with Glutathione Peroxidase Assay Kit (ab102350, Abcam) according to the manufacturer’s instructions. Approximately 4 × 10^5^ cells/well were plated in six-well plates 3 days followed by drug administration. Cells were digested with trypsin and resuspended with assay buffer and centrifuged at 10,000 × *g* for 15 min at 4 °C. The supernatant was used for the assay. After adding NADPH, glutathione reductase solution, and glutathione solutions, mix well and incubate at room temperature for 15 min to deplete all GSSG in the samples. Add cumene hydroperoxide solution to start the GPx reaction. Measure output on a microplate reader at OD340 nm once every 5 min for 40 min. Calculate the concentration of GPx representing activity.

### TEM assay

Mice were cardiac perfused rapidly with ice-cold PBS followed by 4% PFA. Brain tissue was cut into 1 mm brain slices using a mouse coronal slice mold, and then extracted the left CC ~1 mm^3^ from the site of 0.5 mm behind the bregma. All specimens were immersion-fixed in cold 2.5% glutaraldehyde (Top0394, Biotopped, China) for 24 h, followed by 0.1 M PB three times. And then samples were post-fixed in 1% osmium tetroxide (00308, TCI, Japan) for 2 h, followed by 0.1 M PB three times. Samples were dehydrated in ethanol and acetone. After embedding, samples were cut into 50–70 nm sections and placed on copper slot grids, and stained with uranyl acetate (SPI-02624, HEAD BIO, China) and lead citrate (HD17800, HEAD BIO). Images were captured by TEM (JEM-1400plus). All axons in the field are divided into normal and defected myelin according to their morphology and ten axons with different diameters were randomly selected from each field for G-ratio statistics. Over 50 fibers were acquired in randomly selected areas within the CC from each section at a magnification of ×15,000 and analyzed with Image J by an investigator blinded to experimental groups.

### Inductive coupled plasma-mass spectrometry

ICP-MS was used to quantify iron levels in the brain. Dissected tissue was frozen and weighed prior to ICP-MS analysis. Approximately 0.15 g ipsilateral brain tissue from sham or IVH mice was weighed in quartz tubes, combined with 0.80 mL nitric acid. After predigestion at room temperature for 2 h, we placed the quartz tubes in a microwave digestion system (Ultra WAVE, Milestone, Italy). The temperature settings were as follows: room temperature—150 °C (5 min), 150–190 °C (5 min), 190–190 °C (20 min). Obtained solutions were diluted to 8 ml with ultra-pure water (18 MΩ cm) and Indium was added as an internal standard prior to analysis. The samples were analyzed by ICP-MS (ELAN DRC II, PerkinElmer, USA) instrumental parameters were as follows: nebulizer gas flow rate was 1.03 L/min, the auxiliary gas flow was 1.86 L/min, plasma gas flow was 18.0 L/min, RF power 1150 w, dwell time was 50–100 ms, single point peak hopping, mass Resolution was 0.7–0.9 amu.

### Hindlimb placing test

We tested each mouse for Hindlimb placing test on days 1, 3, 7, 14, and 28 post IVH as described previously [14]. The mice were placed on the edge of the table, and the contralateral hind limbs were gently pulled below the edge of the table to observe the retraction of the animal limbs. Quickly retract and place on the edge of the table accurately, scored 0 point; delayed pullback, scored 1 point; no retraction of the limbs, scored 2 points. A total of 10 successful experiments were performed and the total score was recorded for each mouse.

### Gait analysis

Gait analysis was performed using the Catwalk gait analysis system (Noldus Information Technology, The Netherlands) as described previously [54, 55]. In brief, the mice were placed in the dark experimental environment for 0.5 h for adaption before each experiment. At the beginning of the trial, the mouse was placed individually on the walkway and walked freely traverse from one side to another of the walkway glass plate where has a goal box. The videos of footprint images were recorded by a camera positioned under the walkway. The records were converted into digital signatures and processed using CatWalk XT 10.6 software. All mice completed three times of runs with an interval of 10 min. Mice were trained for 3 days prior to the record of baseline (the day before surgery), and the gait was assessed at −1 (baseline), 14, and 28 days after IVH/sham surgery.

### Statistical analysis

Data are presented as mean ± SD, all statistical analyses were carried out with GraphPad Prism 7.0 Software. *P* values were calculated with a two-tailed Student’s unpaired *t* test for two groups. One-way analysis of variance (ANOVA) was used for comparisons within multiple groups. Two-way ANOVA was used to evaluate groups differentially regulated by the time factor. Tukey’s post hoc test or Dunnett’s post hoc test was used to determine specific differences between groups. *P* values of <0.05 indicated statistically significant differences.

## Supplementary information

Supplementary figure and figure legends

Supplementary material-original western blots

Co-author email responses to authorship changes

change of authorship agreement

checklist

## Data Availability

All data are available in the main text or supplementary materials.
