# Supplementary material for: Ferroptosis in oligodendrocyte progenitor cells mediates white matter injury after hemorrhagic stroke
Source: Cell Death Dis. 2022 Mar 23;13(3):259. doi: 10.1038/s41419-022-04712-0 (PMC8941078; doi:10.1038/s41419-022-04712-0)
Supplement: Supplementary file 1 — Supplementary figure and figure legends [file 41419_2022_4712_MOESM1_ESM.docx]

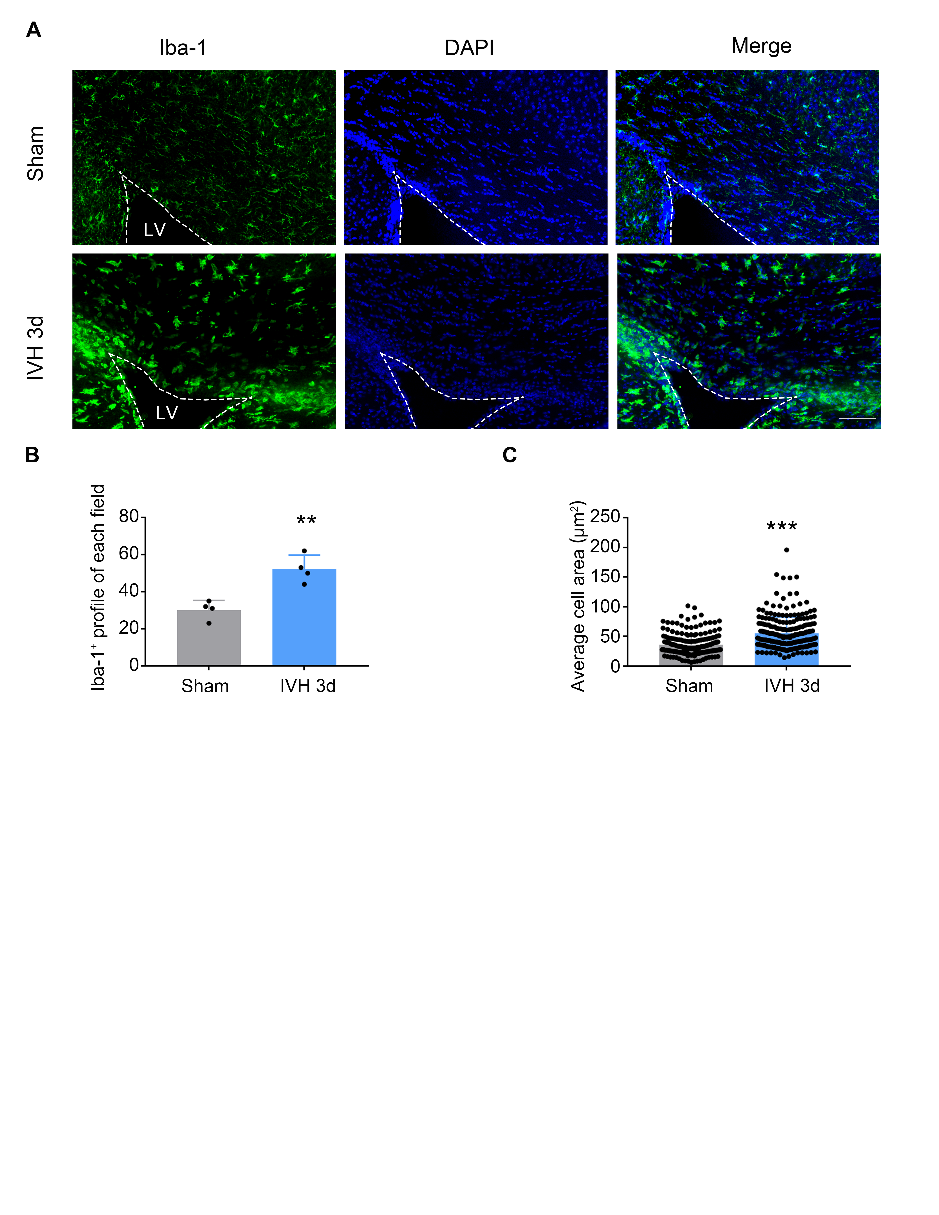


**Figure s1. Microglia activation in IVH mouse brains.**Sham and IVH animal brain slices were immunostained with Iba-1 and DAPI at 3 days post-surgery. (**A**) Representative images are shown at the ipsilateral peri-lateral vesicular regions. (**B**) The profile of Iba-1^+^ cells was calculated. (**C**) The body area of Iba-1^+^ cells was quantified. Results are presented as scatter plots (mean ±SD). B, C: Two-tailed Student’s *t*-test followed by Welch’s correction. ***p*<0.01, ****p*<0.001 vs. sham; Each group contained 4 (B, C) animals. 209 and 234 Iba-1^+^ cells of sham and IVH group were quantified in C. Scale bars: (A) 100 μm. Experiments were repeated at least 3 times independently.

**
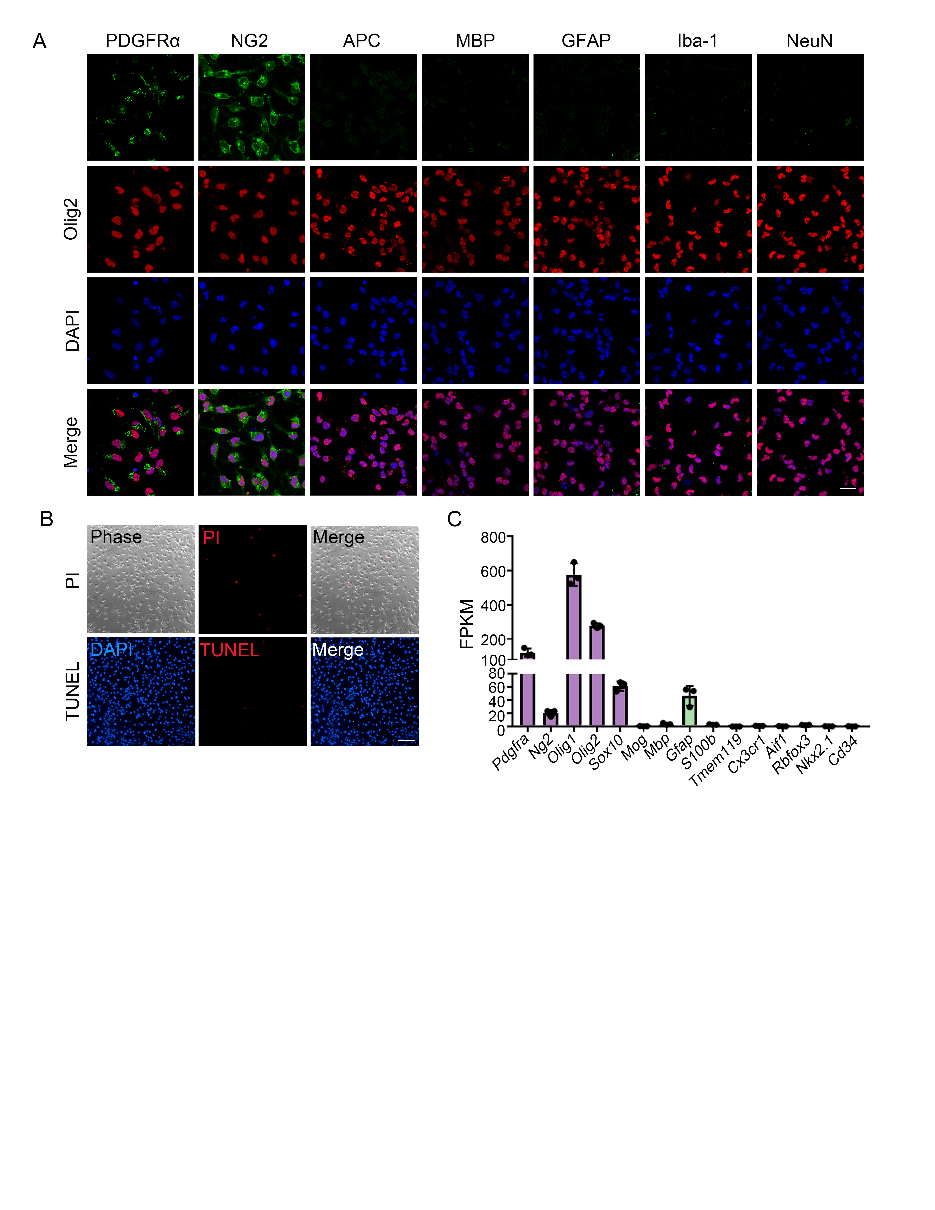
**

**Figure s2. Purity and viability of primary OPC cultures.** (**A**) Immunostaining using PDGFRα, NG2, APC, MBP, GFAP, Iba-1, or NeuN with Olig2 and DAPI on day 3 after dissociating mouse primary OPCs. (**B**) Representative images of PI^+^ and TUNEL^+^ cells are shown. (**C**) RNA-seq analysis was performed and gene expression in vehicle-treated OPCs was analyzed. Experiments were repeated at least 3 times independently. Scale bars: (A) 20 μm; (B) 100 μm.


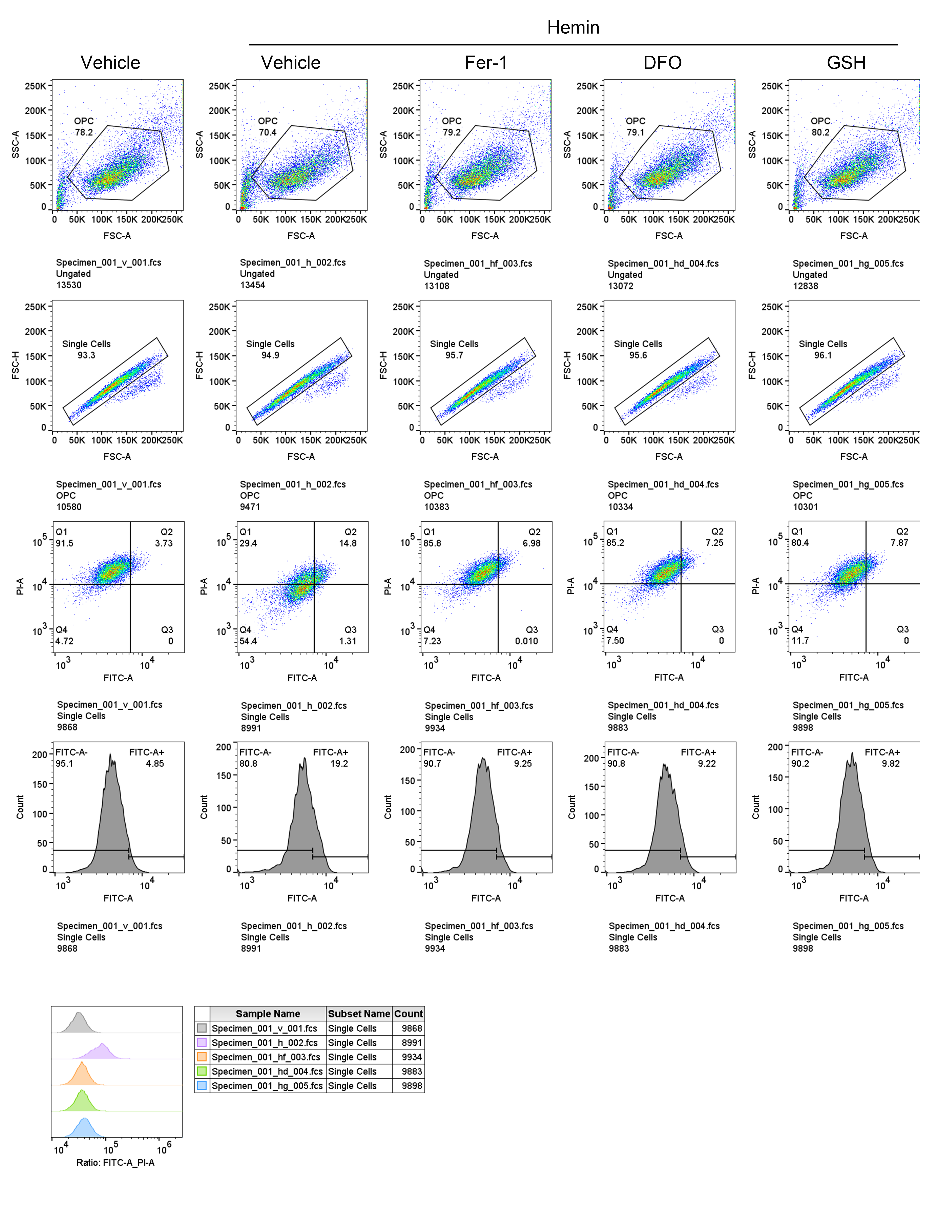


**Figure s3. Hemin-induces lipid ROS in OPCs *in vitro*.** Gate strategy and original plots for lipid ROS detection using OPCs treated as indicated.


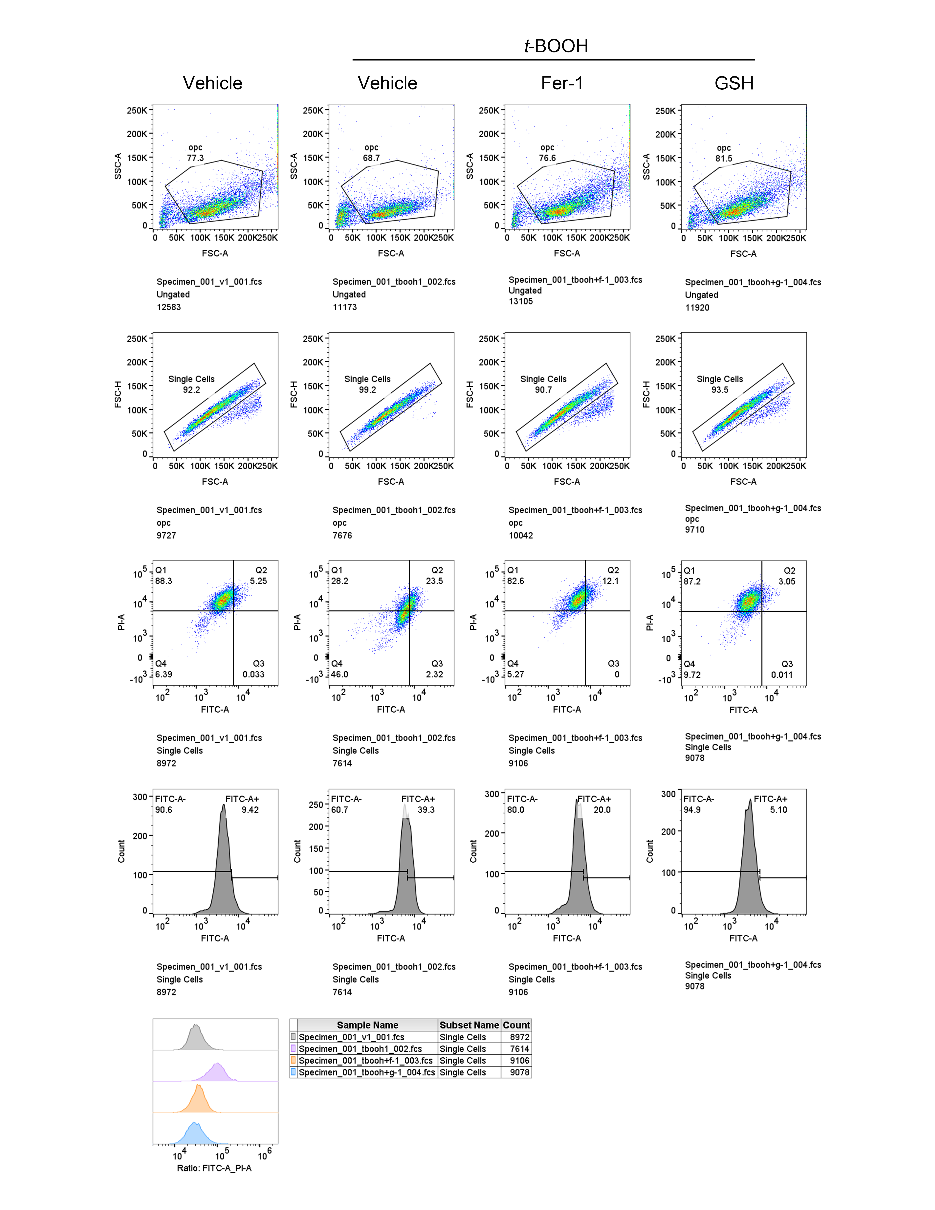


**Figure s4. T-BOOH-induces lipid ROS in OPCs *in vitro*.** Gate strategy and original plots for lipid ROS detection using OPCs treated as indicated.
