# Supplementary figures and images for: Ferroptosis in oligodendrocyte progenitor cells mediates white matter injury after hemorrhagic stroke
Source: Cell Death Dis. 2022 Mar 23;13(3):259. doi: 10.1038/s41419-022-04712-0 (PMC8941078; doi:10.1038/s41419-022-04712-0)

Fig 3.

K

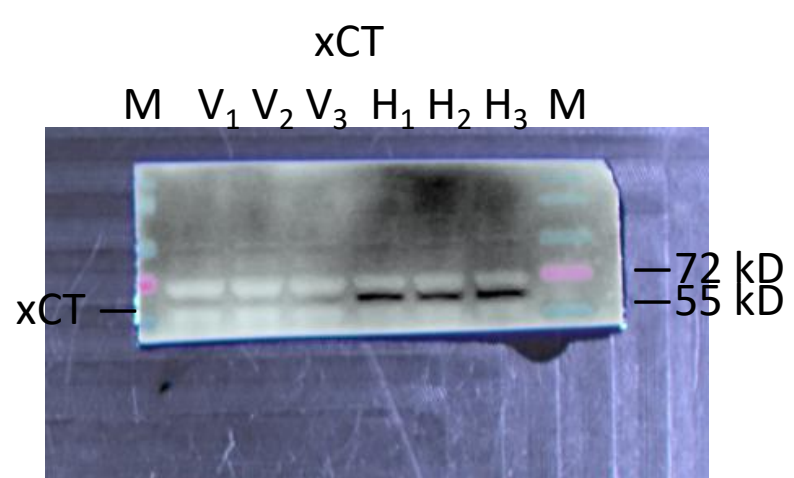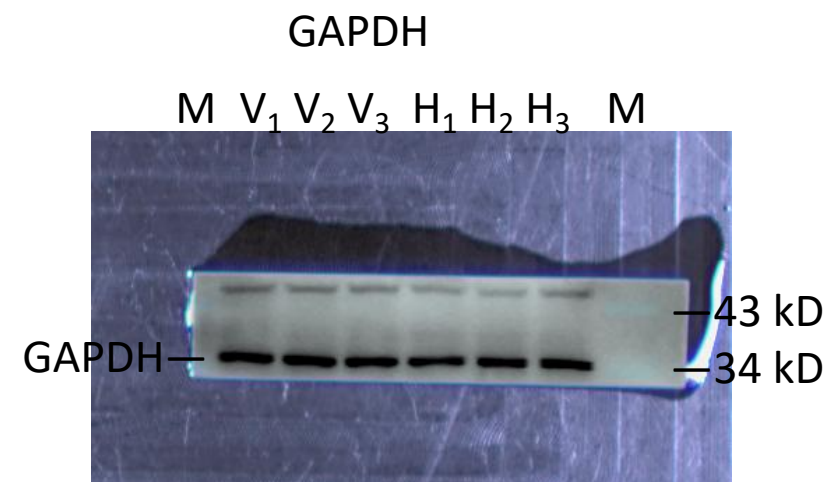

Fig 4.

K

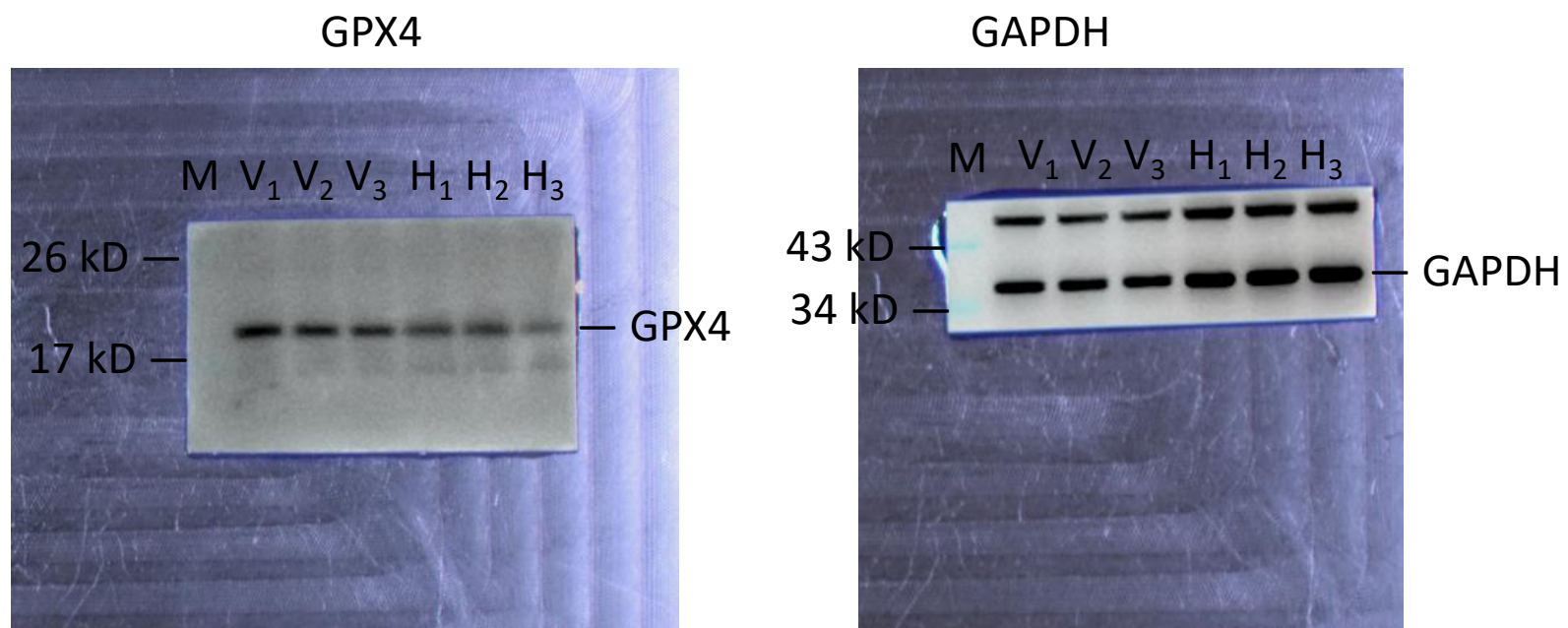

Supplement: Supplementary file 2 — Supplementary material-original western blots [file 41419_2022_4712_MOESM2_ESM.pdf]
