# Supplementary material for: Ferroptosis in oligodendrocyte progenitor cells mediates white matter injury after hemorrhagic stroke
Source: Cell Death Dis. 2022 Mar 23;13(3):259. doi: 10.1038/s41419-022-04712-0 (PMC8941078; doi:10.1038/s41419-022-04712-0)
Supplement: Supplementary file 4 — change of authorship agreement [file 41419_2022_4712_MOESM4_ESM.pdf]

SPRINGER NATURE

Change of authorship request form - Journals (pre-acceptance)

Important information. Please read.

- This form should be used by authors to request any change in authorship (adding/deleting authors) including changes in corresponding authors. This form should not be used for name changes. Please fully complete all sections. Use black ink and block capitals and provide each author's full name with the given name first followed by the family name.

- By signing this declaration, all authors guarantee that the order of the authors are in accordance with their scientific contribution, if applicable as different conventions apply per discipline, and that only authors have been added who made a meaningful contribution to the work.

- Please note, in author collaborations where there is formal agreement for representing the collaboration, it is sufficient for the representative or legal guarantor (usually the corresponding author) to complete and sign the Authorship Change Form on behalf of all authors, **next to the added/removed author(s)**. **(Complete Section 3, followed by Section 6.)**

  In author collaborations where there is no formal agreement for representing the collaboration and **there are more than 10 authors**, one may sign for all, provided the signer appends correspondence that attests that each of the authors have agreed to the change **and the added/removed authors sign the form**. **(Complete Section 3, followed by Section 6.)**

- Please note, we cannot investigate or mediate any authorship disputes. If you are unable to obtain agreement from all authors (including those who you wish to be removed) you must refer the matter to your institution(s) for investigation. Please inform us if you need to do this.

- If you are not able to return a fully completed form within **30 days** of the date that it was sent to the author requesting the change, we may have to withdraw your manuscript. We cannot publish manuscripts where authorship has not been agreed by all authors (including those who have been removed).

- Incomplete forms will be rejected.

- Please return/upload this form, fully completed, to the Journals Editorial Office. The Journal and/or Publisher will consider the information you have provided to decide whether to approve the proposed change in authorship. We may decide to contact your institution for more information or undertake a further investigation, if appropriate, before making a final decision.

SPRINGER NATURE Springer Nature is one of the world's leading global research, educational and professional publishers, created in May 2015 through the combination of Nature Publishing Group, Palgrave Macmillan, Macmillan Education and Springer Science+Business Media.

SPRINGER NATURE

Change of authorship request form - Journals (pre-acceptance)

Section 1: Please provide the current title of manuscript

Manuscript ID no.: CDDIS-21-4247

Title: Ferroptosis in oligodendrocyte progenitor cells mediates white matter injury after hemorrhagic stroke

Section 2: Please provide the previous authorship, in the order shown on the manuscript before the changes were introduced. Please indicate the corresponding author by adding (CA) behind the name.

	First name(s)	Family name	ORCID or SCOPUS id, if available
1st author	Danmin	Shen	0000-0002-3535-0145
2nd author	Weihua	Wu	0000-0002-6060-5453
3rd author	Ting	Lan	0000-0002-4508-7301
4th author	Zhongnan	Xiao	0000-0001-5530-0287
5th author	Kaiyuan	Gai	0000-0002-3640-8329
6th author	Liye	Hu	0000-0003-3843-7913
7th author	Zhaoli	Luo	0000-0003-0372-0479
8th author	Chao	Wei	0000-0003-1959-4980
9th author	Yabin	Lu	0000-000207077-8788
10th author	Yamei	Wang	0000-0002-8240-3991

Please use an additional sheet if there are more than 10 authors.

SPRINGER NATURE Springer Nature is one of the world's leading global research, educational and professional publishers, created in May 2015 through the combination of Nature Publishing Group, Palgrave Macmillan, Macmillan Education and Springer Science+Business Media.

SPRINGER NATURE

Change of authorship request form - Journals (pre-acceptance)

Section 1: Please provide the current title of manuscript

Manuscript ID no.: CDDIS-21-4247

Title: Ferroptosis in oligodendrocyte progenitor cells mediates white matter injury after hemorrhagic stroke

Section 2: Please provide the previous authorship, in the order shown on the manuscript before the changes were introduced. Please indicate the corresponding author by adding (CA) behind the name.

	First name(s)	Family name	ORCID or SCOPUS id, if available
11st author	Chenguang	Zhang	0000-0003-4093-9322
12nd author	Peipei	Wang	0000-0002-9704-539X
13rd author	Zhentao	Zuo	0000-0002-5909-3884
14th author	Fei	Yang	0000-0003-3122-2960
15th author	Qian	Li (CA)	0000-0003-0359-5604
16th author			
17th author			
18th author			
19th author			
20th author			

Please use an additional sheet if there are more than 10 authors.

SPRINGER NATURE Springer Nature is one of the world’s leading global research, educational and professional publishers, created in May 2015 through the combination of Nature Publishing Group, Palgrave Macmillan, Macmillan Education and Springer Science+Business Media.

SPRINGER NATURE

Change of authorship request form - Journals (pre-acceptance)

Section 3: Please provide a justification for change. Please use this section to explain your reasons for changing the authorship of your manuscript, e.g. what necessitated the change in authorship? Please refer to the (journal) policy pages for more information about authorship. Please explain why omitted authors were not originally included and/or why authors were removed on the submitted manuscript.

We apply to add Liu Jing and Xiaotong Wang as co authors to affirm their contribution to the experimental part during the revision of the article.

Section 4: Proposed new authorship. Please provide your new authorship list in the order you would like it to appear on the manuscript. Please indicate the corresponding author by adding (CA) behind the name. <u>If the Corresponding Author has changed, please indicate the reason under section 3</u>.

	First name(s)	Family name (this name will appear in full on the final publication and will be searchable in various abstract and indexing databases)	Affiliated institute	E-mail address
1st author	Danmin	Shen	Department of Biochemistry and Molecular Biology, School of Basic Medical Sciences, Capital Medical University, Beijing, 100069, China	15810756234@163.com
2nd author	Weihua	Wu	Department of Biochemistry and Molecular Biology, School of Basic Medical Sciences, Capital Medical University, Beijing, 100069, China	wuweihua@mail.ccmu.edu.cn
3rd author	Jing	Liu	Department of Neurobiology, School of Basic Medical Sciences, Capital Medical University, Beijing, 100069, China	jingliu8762@163.com
4th author	Ting	Lan	Department of Biochemistry and Molecular Biology, School of Basic Medical Sciences, Capital Medical University, Beijing, 100069, China	tinglanzs@163.com

---
SPRINGER NATURE Springer Nature is one of the world's leading global research, educational and professional publishers, created in May 2015 through the combination of Nature Publishing Group, Palgrave Macmillan, Macmillan Education and Springer Science+Business Media.

5th author	Zhongnan	Xiao	Department of Biochemistry and Molecular Biology, School of Basic Medical Sciences, Capital Medical University, Beijing, 100069, China	xzn1016@126.com
6th author	Kaiyuan	Gai	School of Basic Medical Sciences, Capital Medical University, Beijing, 100069, China	gaikaiyuan2000@163.com
7th author	Liye	Hu	Department of Biochemistry and Molecular Biology, School of Basic Medical Sciences, Capital Medical University, Beijing, 100069, China	huliye@mail.ccmu.edu.cn
8th author	Zhaoli	Luo	Department of Biochemistry and Molecular Biology, School of Basic Medical Sciences, Capital Medical University, Beijing, 100069, China	zhaoliluo@mail.ccmu.edu.cn
9th author	Chao	Wei	Department of Neurobiology, School ofBasic Medical Sciences, Capital Medical University, Beijing, 100069, China	chaowei@ccmu.edu.cn
10th author	Xiaotong	Wang	Department of Neurobiology, School ofBasic Medical Sciences, Capital Medical University, Beijing, 100069, China	wangxt1414@163.com
11st author	Yabin	Lu	Department of Biochemistry and Molecular Biology, School of Basic Medical Sciences, Capital Medical University, Beijing, 100069, China	luyabin@ccmu.edu.cn
12nd author	Yamei	Wang	Department of Biochemistry and Molecular Biology, School of Basic Medical Sciences, Capital Medical University, Beijing, 100069, China	ymwang@ccmu.edu.cn
13rd author	Chenguang	Zhang	Department of Biochemistry and Molecular Biology, School of Basic Medical Sciences, Capital Medical University, Beijing, 100069, China	chzhang@ccmu.edu.cn
14th author	Peipei	Wang	Department of Neurobiology, School of Basic Medical Sciences, Capital Medical University, Beijing, 100069, China	wangpp@ccmu.edu.cn

15th author	Zhentao	Zuo	State Key Laboratory of Brain and Cognitive Science, Institute of Biophysics, Chinese Academy of Sciences, Beijing, 100101, China; Hefei Comprehensive National Science Center, Institute of Artificial Intelligence, Hefei, China; Sino-Danish College, University of Chinese Academy of Sciences, Beijing 100049, China	zuozt@ibp.ac.cn
16th author	Fei	Yang	Department of Neurobiology, School of Basic Medical Sciences, Capital Medical University, Beijing, 100069, China; Advanced Innovation Center for Human Brain Protection, Beijing Key Laboratory of Neural Regeneration and Repair, Capital Medical University, Beijing, 100069, China	feiyang@ccmu.edu.cn
17th author	Qian	Li (CA)	Department of Biochemistry and Molecular Biology, School of Basic Medical Sciences, Capital Medical University, Beijing, 100069, China; Advanced Innovation Center for Human Brain Protection, Beijing Key Laboratory of Neural Regeneration and Repair, Capital Medical University, Beijing, 100069, China	qianli@ccmu.edu.cn

Please use an additional sheet if there are more than 10 authors.

SPRINGER NATURE Springer Nature is one of the world's leading global research, educational and professional publishers, created in May 2015 through the combination of Nature Publishing Group, Palgrave Macmillan, Macmillan Education and Springer Science+Business Media.

SPRINGER NATURE

Change of authorship request form - Journals (pre-acceptance)

Section 5: Author contribution, Acknowledgement and Disclosures. Please use this section to provide a new disclosure statement and, if appropriate, acknowledge any contributors who have been removed as authors and ensure you state what contribution any new authors made (if applicable per the journal or book (series) policy). Please ensure these are updated in your manuscript - after approval of the change(s) - as our production department will not transfer the information in this form to your manuscript.

New acknowledgements:

Conceptualization: DS, QL
Data curation: DS, WW, JL, ZX, KG, CW, XW, FY, QL
Formal analysis: DS, WW, TL, CZ, KG, LH, ZL, CW, ZZ, FY, QL
Methodology: DS, WW, ZZ, QL
Investigation: DS, WW, TL, CZ, KG, LH, ZL, CW, FY, QL
Visualization: DS, ZZ, QL
Funding acquisition: ZZ, FY, QL
Project administration: PW, YW, YL, FY, QL
Supervision: FY, QL
Writing – original draft: DS, QL
Writing – review & editing: DS, YL, CZ, FY, QL

New Disclosures (financial and non-financial interests, funding):

Not changeable.

New Author Contributions statement (if applicable per the journal policy):

We apply to add Liu Jing and Wang Xiaotong as co authors to affirm their contribution to the experimental part during the revision of the article, and increase the contribution of Jing Liu, Chao Wei and Xiaotong Wang in data curation.

State 'Not applicable' if there are no new authors.

SPRINGER NATURE Springer Nature is one of the world's leading global research, educational and professional publishers, created in May 2015 through the combination of Nature Publishing Group, Palgrave Macmillan, Macmillan Education and Springer Science+Business Media.

SPRINGER NATURE

Change of authorship request form - Journals (pre-acceptance)

Section 6: Declaration of agreement. *All* authors, unchanged, new and removed *must* sign this declaration.
(NB: Please print the form, (docu)-sign and return/upload a scanned copy. Please note that signatures that have been inserted as an image file are acceptable as long as it is handwritten. Typed names in the signature box are unacceptable.) *** Please delete as appropriate. Delete all of the bold if you were on the original authorship list and are remaining as an author.**

	First name	Family name		Signature	Date
1st author	Danmin	Shen	I agree to the proposed new authorship shown in section 4 **/and the addition/removal*of my name to the authorship list** /and the proposed change in corresponding author	Danmin Shen	2022.1.28
2nd author	Weihua	Wu	I agree to the proposed new authorship shown in section 4 **/and the addition/removal*of my name to the authorship list** /and the proposed change in corresponding author	Weihua Wu	2022.1.28
3rd author	Jing	Liu	I agree to the proposed new authorship shown in section 4 **/and the addition/removal*of my name to the authorship list** /and the proposed change in corresponding author	Jing Liu	2022.1.28
4th authors	Ting	Lan	I agree to the proposed new authorship shown in section 4 **/and the addition/removal*of my name to the authorship list** /and the proposed change in corresponding author	Ting lan	2022.1.28

SPRINGER NATURE Springer Nature is one of the world's leading global research, educational and professional publishers, created in May 2015 through the combination of Nature Publishing Group, Palgrave Macmillan, Macmillan Education and Springer Science+Business Media.

Change of authorship request form - Journals (pre-acceptance)

5th author	Zhongnan	Xiao	I agree to the proposed new authorship shown in section 4 **/and the addition/removal*of my name to the authorship list** /and the proposed change in corresponding author	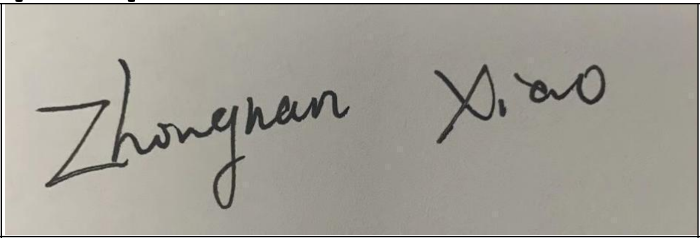	2022.1.28
6th author	Kaiyuan	Gai	I agree to the proposed new authorship shown in section 4 **/and the addition/removal*of my name to the authorship list** /and the proposed change in corresponding author	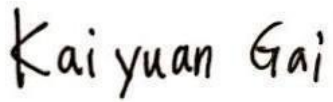	2022.1.28
7th author	Liye	Hu	I agree to the proposed new authorship shown in section 4 **/and the addition/removal*of my name to the authorship list** /and the proposed change in corresponding author	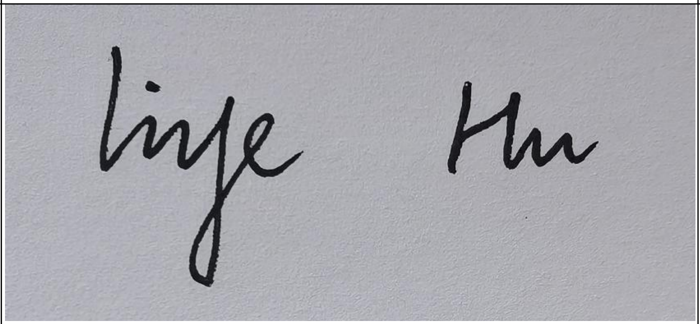	2022.1.28

SPRINGER NATURE Springer Nature is one of the world's leading global research, educational and professional publishers, created in May 2015 through the combination of Nature Publishing Group, Palgrave Macmillan, Macmillan Education and Springer Science+Business Media.

Change of authorship request form - Journals (pre-acceptance)

	First name	Family name		Signature	Date
8th author	Zhaoli	Luo	I agree to the proposed new authorship shown in section 4 **/and the addition/removal*of my name to the authorship list** /and the proposed change in corresponding author	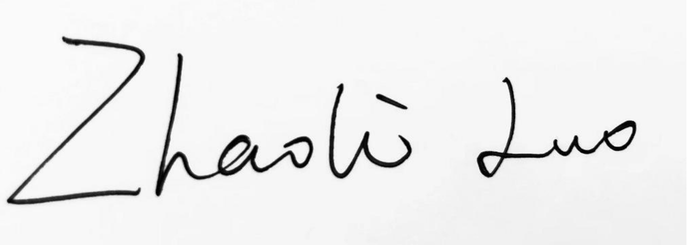	2022.1.28
9th author	Chao	Wei	I agree to the proposed new authorship shown in section 4 **/and the addition/removal*of my name to the authorship list** /and the proposed change in corresponding author	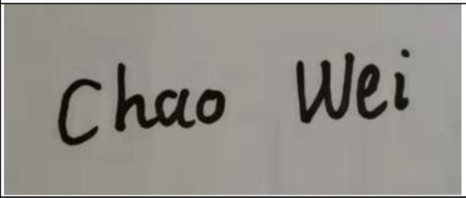	2022.1.28
10th author	Xiaotong	Wang	I agree to the proposed new authorship shown in section 4 **/and the addition/removal*of my name to the authorship list** /and the proposed change in corresponding author	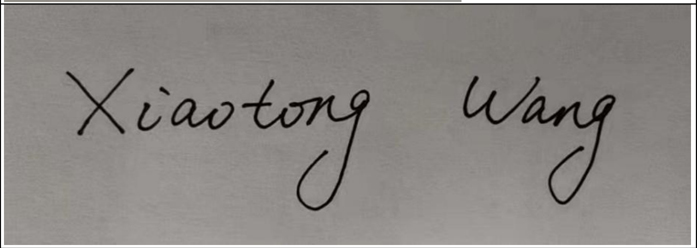	2022.1.28
11th author	Yabin	Lu	I agree to the proposed new authorship shown in section 4 **/and the addition/removal*of my name to the authorship list** /and the proposed change in corresponding author	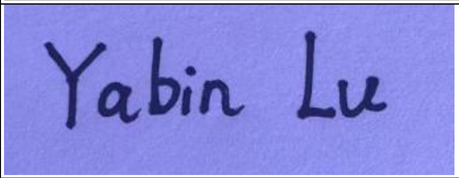	2022.1.28

SPRINGER NATURE Springer Nature is one of the world's leading global research, educational and professional publishers, created in May 2015 through the combination of Nature Publishing Group, Palgrave Macmillan, Macmillan Education and Springer Science+Business Media.

12th author	Yamei	Wang	I agree to the proposed new authorship shown in section 4 **/and the addition/removal*of my name to the authorship list** /and the proposed change in corresponding author	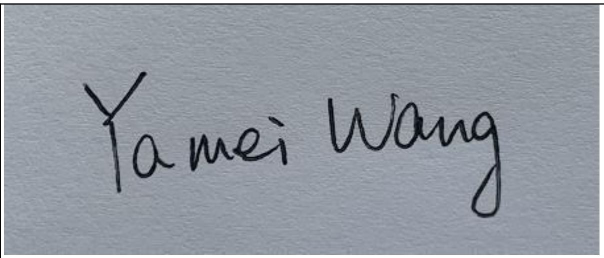	2022.1.28
13th author	Chenguang	Zhang	I agree to the proposed new authorship shown in section 4 **/and the addition/removal*of my name to the authorship list** /and the proposed change in corresponding author	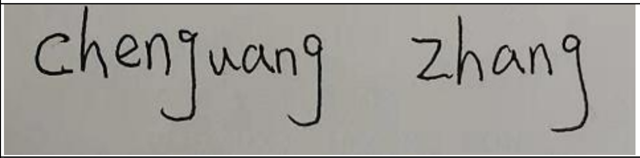	2022.1.28
14th author	Peipei	Wang	I agree to the proposed new authorship shown in section 4 **/and the addition/removal*of my name to the authorship list** /and the proposed change in corresponding author	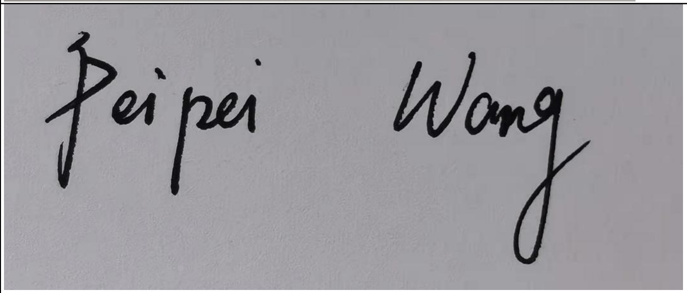	2022.1.28
15th author	Zhentao	Zuo	I agree to the proposed new authorship shown in section 4 **/and the addition/removal*of my name to the authorship list** /and the proposed change in corresponding author	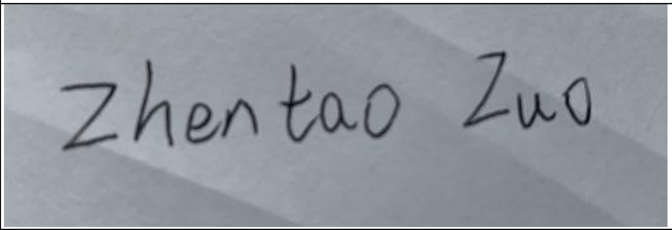	2022.1.28
16th author	Fei	Yang	I agree to the proposed new authorship shown in section 4 **/and the addition/removal*of my name to the authorship list** /and the proposed change in corresponding author	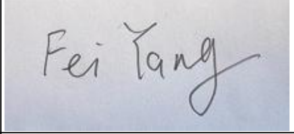	2022.1.28
17th author	Qian	Li	I agree to the proposed new authorship shown in section 4 **/and the addition/removal*of my name to the authorship list** /and the proposed change in corresponding author	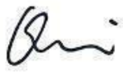	2022.1.28

SPRINGER NATURE Springer Nature is one of the world's leading global research, educational and professional publishers, created in May 2015 through the combination of Nature Publishing Group, Palgrave Macmillan, Macmillan Education and Springer Science+Business Media.

SPRINGER NATURE

Change of authorship request form - Journals (pre-acceptance)

Please use an additional sheet if there are more than 10 authors.

In case of author collaborations with formal agreement:

	Name of consortium/consortia	First name	Family name		Signature	Date
Representative/legal guarantor				I agree to the proposed new authorship shown in section 4 /**and the addition/removal*of my name to the authorship list** /and the proposed change in corresponding author		

Both added/removed authors should complete the information in the first table under Section 6.

---- End of form ----

SPRINGER NATURE Springer Nature is one of the world's leading global research, educational and professional publishers, created in May 2015 through the combination of Nature Publishing Group, Palgrave Macmillan, Macmillan Education and Springer Science+Business Media.
