# Supplementary material for: Ferroptosis in oligodendrocyte progenitor cells mediates white matter injury after hemorrhagic stroke
Source: Cell Death Dis. 2022 Mar 23;13(3):259. doi: 10.1038/s41419-022-04712-0 (PMC8941078; doi:10.1038/s41419-022-04712-0)
Supplement: Supplementary file 3 — Co-author email responses to authorship changes [file 41419_2022_4712_MOESM3_ESM.pdf]

Subject:	Re:Change of Attribution Agreement	
From:	"沈丹敏" <15810756234@163.com>	Feb 22, 2022 6:46:30 PM
To:	"李纤" <qianli@ccmu.edu.cn>	

I agree.

Danmin Shen

At 2022-02-22 18:25:15, "李纤" <qianli@ccmu.edu.cn> wrote:

> Dear co-authors,
>
> As you know, we have added Dr. Jing Liu and Ms. Xiaotong Wang as coauthors to this manuscript entitled "Ferroptosis in oligodendrocyte progenitor cells mediates white matter injury after hemorrhagic stroke". Please reply to my email and confirm that you have agreed with these changes.
>
> Best wishes,
>
> Qian Li
>
> ----------------
>
> Dear Professor Li,
>
> In checking in your manuscript submitted to Cell Death & Disease it has come to our attention that the following must be addressed before we can proceed.
>
> 1. It has come to our attention that your most recent author list differs from the one in your original submission.
>
> Please request agreement from all authors including additions and deletions, these can be collected in the following way:
>
> Email your co-authors with the change, and ask them to reply to your email confirming that they agree to these changes. Once you have collected these replies, please combine all of the co-authors' email responses in one document and upload this file to your submission.
>
> Your paper is now available for you to edit, you may access via the following link:

https://mts-cddis.nature.com/cgi-bin/main.plex?el=A7CA3WBn4C6BzWt2F4A9ftdnKFtOtGprSVjttlqtJVQZ

(Press/Click on the above link to be automatically sent to the web page.)

Please make the correction(s) as specified above and resubmit your paper following the same steps as before.

If you have any questions please feel free to contact us.

Sincerely,

Editorial Office
Cell Death & Disease
cddisease@springernature.com

Subject:	Re: Change of Attribution Agreement
From:	"吴伟华" <wuweihua@mail.ccmu.edu.cn> Feb 22, 2022 6:34:24 PM
To:	"李纤" <qianli@ccmu.edu.cn>

I agree.
-Weihua Wu.

-----原始邮件-----
发件人:"李纤" <qianli@ccmu.edu.cn>
发送时间:2022-02-22 18:25:15 (星期二)
收件人: 15810756234@163.com, wuweihua@mail.ccmu.edu.cn, jingliu8762@163.com, tinglanzs@163.com, xzn1016@126.com, gaikaiyuan2000@163.com, huliye@mail.ccmu.edu.cn, zhaoliluo@mail.ccmu.edu.cn, chaowei@ccmu.edu.cn, wangxt1414@163.com, luyabin@ccmu.edu.cn, ymwang@ccmu.edu.cn, chzhang@ccmu.edu.cn, wangpp@ccmu.edu.cn, zuozt@ibp.ac.cn, feiyang@ccmu.edu.cn
抄送: qianli@ccmu.edu.cn
主题: Change of Attribution Agreement

Dear co-authors,

As you know, we have added Dr. Jing Liu and Ms. Xiaotong Wang as coauthors to this manuscript entitled "Ferroptosis in oligodendrocyte progenitor cells mediates white matter injury after hemorrhagic stroke". Please reply to my email and confirm that you have agreed with these changes.

Best wishes,

Qian Li

Dear Professor Li,

In checking in your manuscript submitted to Cell Death & Disease it has come to our attention that the following must be addressed before we can proceed.

1. It has come to our attention that your most recent author list differs from the one in your original submission.

Please request agreement from all authors including additions and deletions, these can be collected in the following way:

Email your co-authors with the change, and ask them to reply to your email confirming that they agree to these changes. Once you have collected these replies, please combine all of the co-authors' email responses in one document and upload this file to your submission.

Your paper is now available for you to edit, you may access via the following link:

https://mts-cddis.nature.com/cgi-bin/main.plex?el=A7CA3WBn4C6BzWt2F4A9ftdnKFtOtGprSVjttlqtJVQZ

(Press/Click on the above link to be automatically sent to the web page.)

Please make the correction(s) as specified above and resubmit your paper following the same steps as before.

If you have any questions please feel free to contact us.

Sincerely,

Editorial Office

Cell Death & Disease

cddisease@springernature.com

Subject:	Re:Change of Attribution Agreement	
From:	"Jing Liu" <jingliu8762@163.com>	Feb 22, 2022 6:37:16 PM
To:	"李纤" <qianli@ccmu.edu.cn>	

Dear Dr. Li,

I agree with these changes.

Best,

Jing Liu

Jing Liu

jingliu8762@163.com

签名由 网易邮箱大师 定制

On 02/22/2022 18:25，李纤<qianli@ccmu.edu.cn> wrote：

Dear co-authors,

As you know, we have added Dr. Jing Liu and Ms. Xiaotong Wang as coauthors to this manuscript entitled "Ferroptosis in oligodendrocyte progenitor cells mediates white matter injury after hemorrhagic stroke". Please reply to my email and confirm that you have agreed with these changes.

Best wishes,

Qian Li

Dear Professor Li,

In checking in your manuscript submitted to Cell Death & Disease it has come to our attention that the following must be addressed before we can proceed.

1. It has come to our attention that your most recent author list differs from the one in your original submission.

Please request agreement from all authors including additions and deletions, these can be collected in the following way:

Email your co-authors with the change, and ask them to reply to your email confirming that they agree to these changes. Once you have collected these replies, please combine all of the co-authors' email responses in one document and upload this file to your submission.

Your paper is now available for you to edit, you may access via the following link:

https://mts-cddis.nature.com/cgi-bin/main.plex?el=A7CA3WBn4C6BzWt2F4A9ftdnKFtOtGprSVjttlqtJVQZ

(Press/Click on the above link to be automatically sent to the web page.)

Please make the correction(s) as specified above and resubmit your paper following the same steps as before.

If you have any questions please feel free to contact us.

Sincerely,

Editorial Office
Cell Death & Disease
cddisease@springernature.com

Subject:	Re: Change of Attribution Agreement
From:	lan <tinglanzs@163.com> Feb 22, 2022 6:32:11 PM
To:	qianli <qianli@ccmu.edu.cn>

I agree.
Ting Lan

tinglanzs

邮箱：tinglanzs@163.com

---- 回复的原邮件 ----

发件人 李纤<qianli@ccmu.edu.cn>

日期 2022年02月22日 18:25

收件人 15810756234@163.com<15810756234@163.com> wuweihua@mail.ccmu.edu.cn<wuweihua@mail.ccmu.edu.cn> jingliu8762@163.com<jingliu8762@163.com> tinglanzs@163.com<tinglanzs@163.com> xzn1016@126.com<xzn1016@126.com> gaikaiyuan2000@163.com<gaikaiyuan2000@163.com> huliye@mail.ccmu.edu.cn<huliye@mail.ccmu.edu.cn> zhaoliluo@mail.ccmu.edu.cn<zhaoliluo@mail.ccmu.edu.cn> chaowei@ccmu.edu.cn<chaowei@ccmu.edu.cn> wangxt1414@163.com<wangxt1414@163.com> luyabin@ccmu.edu.cn<luyabin@ccmu.edu.cn> ymwang@ccmu.edu.cn<ymwang@ccmu.edu.cn> chzhang@ccmu.edu.cn<chzhang@ccmu.edu.cn> wangpp@ccmu.edu.cn<wangpp@ccmu.edu.cn> zuozt@ibp.ac.cn<zuozt@ibp.ac.cn> feiyang@ccmu.edu.cn<feiyang@ccmu.edu.cn>

抄 送 至 qianli@ccmu.edu.cn<qianli@ccmu.edu.cn>

主 题 Change of Attribution Agreement

Dear co-authors,

As you know, we have added Dr. Jing Liu and Ms. Xiaotong Wang as coauthors to this manuscript entitled "Ferroptosis in oligodendrocyte progenitor cells mediates white matter injury after hemorrhagic stroke". Please reply to my email and confirm that you have agreed with these changes.

Best wishes,

Qian Li

Dear Professor Li,

In checking in your manuscript submitted to Cell Death & Disease it has come to our attention that the following must be addressed before we can proceed.

1. It has come to our attention that your most recent author list differs from the one in your original submission.

Please request agreement from all authors including additions and deletions, these can be collected in the

following way:

Email your co-authors with the change, and ask them to reply to your email confirming that they agree to these changes. Once you have collected these replies, please combine all of the co-authors' email responses in one document and upload this file to your submission.

Your paper is now available for you to edit, you may access via the following link:

https://mts-cddis.nature.com/cgi-bin/main.plex?el=A7CA3WBn4C6BzWt2F4A9ftdnKFtOtGprSVjttlqtJVQZ

(Press/Click on the above link to be automatically sent to the web page.)

Please make the correction(s) as specified above and resubmit your paper following the same steps as before.

If you have any questions please feel free to contact us.

Sincerely,

Editorial Office
Cell Death & Disease
cddisease@springernature.com

Subject:	Re:Change of Attribution Agreement
From:	"肖中男" <xzn1016@126.com> Feb 22, 2022 6:43:23 PM
To:	"李纤" <qianli@ccmu.edu.cn>

I agree.

Zhongnan Xiao

At 2022-02-22 18:25:15, "李纤" <qianli@ccmu.edu.cn> wrote:

> Dear co-authors,
>
> As you know, we have added Dr. Jing Liu and Ms. Xiaotong Wang as coauthors to this manuscript entitled "Ferroptosis in oligodendrocyte progenitor cells mediates white matter injury after hemorrhagic stroke". Please reply to my email and confirm that you have agreed with these changes.
>
> Best wishes,
>
> Qian Li
>
> ----------------
>
> Dear Professor Li,
>
> In checking in your manuscript submitted to Cell Death & Disease it has come to our attention that the following must be addressed before we can proceed.
>
> 1. It has come to our attention that your most recent author list differs from the one in your original submission.
>
> Please request agreement from all authors including additions and deletions, these can be collected in the following way:
>
> Email your co-authors with the change, and ask them to reply to your email confirming that they agree to these changes. Once you have collected these replies, please combine all of the co-authors' email responses in one document and upload this file to your submission.

Your paper is now available for you to edit, you may access via the following link:

https://mts-cddis.nature.com/cgi-bin/main.plex?el=A7CA3WBn4C6BzWt2F4A9ftdnKFtOtGprSVjttlqtJVQZ

(Press/Click on the above link to be automatically sent to the web page.)

Please make the correction(s) as specified above and resubmit your paper following the same steps as before.

If you have any questions please feel free to contact us.

Sincerely,

Editorial Office
Cell Death & Disease
cddisease@springernature.com

Subject:	Re: Change of Attribution Agreement	
From:	"盖开元" <gaikaiyuan2000@163.com>	Feb 22, 2022 6:29:39 PM
To:	"李纤" <qianli@ccmu.edu.cn>	

I agree. Kaiyuan Gai

---- Replied Message ----

From	李纤<qianli@ccmu.edu.cn>
Date	02/22/2022 18:25
To	15810756234<15810756234@163.com>,<br>wuweihua<wuweihua@mail.ccmu.edu.cn>,<br>jingliu8762<jingliu8762@163.com>,<br>tinglanzs<tinglanzs@163.com>,<br>xzn1016<xzn1016@126.com>,<br>gaikaiyuan2000<gaikaiyuan2000@163.com>,<br>huliye<huliye@mail.ccmu.edu.cn>,<br>zhaoliluo<zhaoliluo@mail.ccmu.edu.cn>,<br>chaowei<chaowei@ccmu.edu.cn>,<br>wangxt1414<wangxt1414@163.com>,<br>luyabin<luyabin@ccmu.edu.cn>,<br>ymwang<ymwang@ccmu.edu.cn>,<br>chzhang<chzhang@ccmu.edu.cn>,<br>wangpp<wangpp@ccmu.edu.cn>,<br>zuozt<zuozt@ibp.ac.cn>,<br>feiyang<feiyang@ccmu.edu.cn>
Cc	qianli<qianli@ccmu.edu.cn>
Subject	Change of Attribution Agreement

Dear co-authors,

As you know, we have added Dr. Jing Liu and Ms. Xiaotong Wang as coauthors to this manuscript entitled "Ferroptosis in oligodendrocyte progenitor cells mediates white matter injury after hemorrhagic stroke". Please reply to my email and confirm that you have agreed with these changes.

Best wishes,

Qian Li

Dear Professor Li,

In checking in your manuscript submitted to Cell Death & Disease it has come to our attention that the following must be addressed before we can proceed.

1. It has come to our attention that your most recent author list differs from the one in your original submission.

Please request agreement from all authors including additions and deletions, these can be collected in the following way:

Email your co-authors with the change, and ask them to reply to your email confirming that they agree to these changes. Once you have collected these replies, please combine all of the co-authors' email responses in one document and upload this file to your submission.

Your paper is now available for you to edit, you may access via the following link:

https://mts-cddis.nature.com/cgi-bin/main.plex?el=A7CA3WBn4C6BzWt2F4A9ftdnKFtOtGprSVjttlqtJVQZ

(Press/Click on the above link to be automatically sent to the web page.)

Please make the correction(s) as specified above and resubmit your paper following the same steps as before.

If you have any questions please feel free to contact us.

Sincerely,

Editorial Office
Cell Death & Disease
cddisease@springernature.com

Subject:	Re: Change of Attribution Agreement
From:	"胡丽叶" <huliye@mail.ccmu.edu.cn> Feb 22, 2022 6:53:30 PM
To:	"李纤" <qianli@ccmu.edu.cn>

I agree. Liye Hu

-----原始邮件-----
发件人:"李纤" <qianli@ccmu.edu.cn>
发送时间:2022-02-22 18:25:15 (星期二)
收件人: 15810756234@163.com, wuweihua@mail.ccmu.edu.cn, jingliu8762@163.com, tinglanzs@163.com, xzn1016@126.com, gaikaiyuan2000@163.com, huliye@mail.ccmu.edu.cn, zhaoliluo@mail.ccmu.edu.cn, chaowei@ccmu.edu.cn, wangxt1414@163.com, luyabin@ccmu.edu.cn, ymwang@ccmu.edu.cn, chzhang@ccmu.edu.cn, wangpp@ccmu.edu.cn, zuozt@ibp.ac.cn, feiyang@ccmu.edu.cn
抄送: qianli@ccmu.edu.cn
主题: Change of Attribution Agreement

Dear co-authors,

As you know, we have added Dr. Jing Liu and Ms. Xiaotong Wang as coauthors to this manuscript entitled "Ferroptosis in oligodendrocyte progenitor cells mediates white matter injury after hemorrhagic stroke". Please reply to my email and confirm that you have agreed with these changes.

Best wishes,

Qian Li

Dear Professor Li,

In checking in your manuscript submitted to Cell Death & Disease it has come to our attention that the following must be addressed before we can proceed.

1. It has come to our attention that your most recent author list differs from the one in your original submission.

Please request agreement from all authors including additions and deletions, these can be collected in the following way:

Email your co-authors with the change, and ask them to reply to your email confirming that they agree to these changes. Once you have collected these replies, please combine all of the co-authors' email responses in one document and upload this file to your submission.

Your paper is now available for you to edit, you may access via the following link:

https://mts-cddis.nature.com/cgi-bin/main.plex?el=A7CA3WBn4C6BzWt2F4A9ftdnKFtOtGprSVjttlqtJVQZ

(Press/Click on the above link to be automatically sent to the web page.)

Please make the correction(s) as specified above and resubmit your paper following the same steps as before.

If you have any questions please feel free to contact us.

Sincerely,

Editorial Office
Cell Death & Disease
cddisease@springernature.com

Subject:	Re: Change of Attribution Agreement
From:	"罗兆莉" <zhaoliluo@mail.ccmu.edu.cn> Feb 22, 2022 6:33:31 PM
To:	"李纤" <qianli@ccmu.edu.cn>

I agree.

Zhaoli Luo

-----原始邮件-----
发件人:"李纤" <qianli@ccmu.edu.cn>
发送时间:2022-02-22 18:25:15 (星期二)
收 件 人 : 15810756234@163.com, wuweihua@mail.ccmu.edu.cn, jingliu8762@163.com, tinglanzs@163.com, xzn1016@126.com, gaikaiyuan2000@163.com, huliye@mail.ccmu.edu.cn, zhaoliluo@mail.ccmu.edu.cn, chaowei@ccmu.edu.cn, wangxt1414@163.com, luyabin@ccmu.edu.cn, ymwang@ccmu.edu.cn, chzhang@ccmu.edu.cn, wangpp@ccmu.edu.cn, zuozt@ibp.ac.cn, feiyang@ccmu.edu.cn
抄送: qianli@ccmu.edu.cn
主题: Change of Attribution Agreement

Dear co-authors,

As you know, we have added Dr. Jing Liu and Ms. Xiaotong Wang as coauthors to this manuscript entitled "Ferroptosis in oligodendrocyte progenitor cells mediates white matter injury after hemorrhagic stroke". Please reply to my email and confirm that you have agreed with these changes.

Best wishes,

Qian Li

Dear Professor Li,

In checking in your manuscript submitted to Cell Death & Disease it has come to our attention that the following must be addressed before we can proceed.

1. It has come to our attention that your most recent author list differs from the one in your original submission.

Please request agreement from all authors including additions and deletions, these can be collected in the following way:

Email your co-authors with the change, and ask them to reply to your email confirming that they agree to these changes. Once you have collected these replies, please combine all of the co-authors' email responses in one document and upload this file to your submission.

Your paper is now available for you to edit, you may access via the following link:

https://mts-cddis.nature.com/cgi-bin/main.plex?el=A7CA3WBn4C6BzWt2F4A9ftdnKFtOtGprSVjttlqtJVQZ

(Press/Click on the above link to be automatically sent to the web page.)

Please make the correction(s) as specified above and resubmit your paper following the same steps as before.

If you have any questions please feel free to contact us.

Sincerely,

Editorial Office
Cell Death & Disease
cddisease@springernature.com

Subject:	Re: Change of Attribution Agreement
From:	"魏超" <chaowei@ccmu.edu.cn> Feb 22, 2022 6:37:51 PM
To:	"李纤" <qianli@ccmu.edu.cn>

I agree

Chao Wei

-----原始邮件-----
发件人:"李纤" <qianli@ccmu.edu.cn>
发送时间:2022-02-22 18:25:15 (星期二)
收件人: 15810756234@163.com, wuweihua@mail.ccmu.edu.cn, jingliu8762@163.com, tinglanzs@163.com, xzn1016@126.com, gaikaiyuan2000@163.com, huliye@mail.ccmu.edu.cn, zhaoliluo@mail.ccmu.edu.cn, chaowei@ccmu.edu.cn, wangxt1414@163.com, luyabin@ccmu.edu.cn, ymwang@ccmu.edu.cn, chzhang@ccmu.edu.cn, wangpp@ccmu.edu.cn, zuozt@ibp.ac.cn, feiyang@ccmu.edu.cn
抄送: qianli@ccmu.edu.cn
主题: Change of Attribution Agreement

Dear co-authors,

As you know, we have added Dr. Jing Liu and Ms. Xiaotong Wang as coauthors to this manuscript entitled "Ferroptosis in oligodendrocyte progenitor cells mediates white matter injury after hemorrhagic stroke". Please reply to my email and confirm that you have agreed with these changes.

Best wishes,

Qian Li

Dear Professor Li,

In checking in your manuscript submitted to Cell Death & Disease it has come to our attention that the following must be addressed before we can proceed.

1. It has come to our attention that your most recent author list differs from the one in your original submission.

Please request agreement from all authors including additions and deletions, these can be collected in the following way:

Email your co-authors with the change, and ask them to reply to your email confirming that they agree to these changes. Once you have collected these replies, please combine all of the co-authors' email responses in one document and upload this file to your submission.

Your paper is now available for you to edit, you may access via the following link:

https://mts-cddis.nature.com/cgi-bin/main.plex?el=A7CA3WBn4C6BzWt2F4A9ftdnKFtOtGprSVjttlqtJVQZ

(Press/Click on the above link to be automatically sent to the web page.)

Please make the correction(s) as specified above and resubmit your paper following the same steps as before.

If you have any questions please feel free to contact us.

Sincerely,

Editorial Office
Cell Death & Disease
cddisease@springernature.com

Subject:	Re:Change of Attribution Agreement
From:	XiaotongWang <wangxt1414@163.com> Feb 22, 2022 6:37:09 PM
To:	"李纤" <qianli@ccmu.edu.cn>

I agree.

Xiaotong Wang

At 2022-02-22 18:25:15, "李纤" <qianli@ccmu.edu.cn> wrote:

> Dear co-authors,
>
> As you know, we have added Dr. Jing Liu and Ms. Xiaotong Wang as coauthors to this manuscript entitled "Ferroptosis in oligodendrocyte progenitor cells mediates white matter injury after hemorrhagic stroke". Please reply to my email and confirm that you have agreed with these changes.
>
> Best wishes,
>
> Qian Li
>
> ----------------
>
> Dear Professor Li,
>
> In checking in your manuscript submitted to Cell Death & Disease it has come to our attention that the following must be addressed before we can proceed.
>
> 1. It has come to our attention that your most recent author list differs from the one in your original submission.
>
> Please request agreement from all authors including additions and deletions, these can be collected in the following way:
>
> Email your co-authors with the change, and ask them to reply to your email confirming that they agree to these changes. Once you have collected these replies, please combine all of the co-authors’ email responses in one

document and upload this file to your submission.

Your paper is now available for you to edit, you may access via the following link:

https://mts-cddis.nature.com/cgi-bin/main.plex?el=A7CA3WBn4C6BzWt2F4A9ftdnKFtOtGprSVjttlqtJVQZ

(Press/Click on the above link to be automatically sent to the web page.)

Please make the correction(s) as specified above and resubmit your paper following the same steps as before.

If you have any questions please feel free to contact us.

Sincerely,

Editorial Office
Cell Death & Disease
cddisease@springernature.com

Subject:	Re: Change of Attribution Agreement
From:	"卢雅彬" <luyabin@ccmu.edu.cn> Feb 22, 2022 7:51:30 PM
To:	"李纤" <qianli@ccmu.edu.cn>

Dear Professor Li,

I agree to add Dr. Jing Liu and Ms. Xiaotong Wang as coauthors to this manuscript entitled "Ferroptosis in oligodendrocyte progenitor cells mediates white matter injury after hemorrhagic stroke"

Best wishes,

Yabin Lu

-----原始邮件-----
发件人:"李纤" <qianli@ccmu.edu.cn>
发送时间:2022-02-22 18:25:15 (星期二)
收 件 人 : 15810756234@163.com, wuweihua@mail.ccmu.edu.cn, jingliu8762@163.com, tinglanzs@163.com, xzn1016@126.com, gaikaiyuan2000@163.com, huliye@mail.ccmu.edu.cn, zhaoliluo@mail.ccmu.edu.cn, chaowei@ccmu.edu.cn, wangxt1414@163.com, luyabin@ccmu.edu.cn, ymwang@ccmu.edu.cn, chzhang@ccmu.edu.cn, wangpp@ccmu.edu.cn, zuozt@ibp.ac.cn, feiyang@ccmu.edu.cn
抄送: qianli@ccmu.edu.cn
主题: Change of Attribution Agreement

Dear co-authors,

As you know, we have added Dr. Jing Liu and Ms. Xiaotong Wang as coauthors to this manuscript entitled "Ferroptosis in oligodendrocyte progenitor cells mediates white matter injury after hemorrhagic stroke". Please reply to my email and confirm that you have agreed with these changes.

Best wishes,

Qian Li

Dear Professor Li,

In checking in your manuscript submitted to Cell Death & Disease it has come to our attention that the following must be addressed before we can proceed.

1. It has come to our attention that your most recent author list differs from the one in your original submission.

Please request agreement from all authors including additions and deletions, these can be collected in the following way:

Email your co-authors with the change, and ask them to reply to your email confirming that they agree to these changes. Once you have collected these replies, please combine all of the co-authors' email responses in one document and upload this file to your submission.

Your paper is now available for you to edit, you may access via the following link:

https://mts-cddis.nature.com/cgi-bin/main.plex?el=A7CA3WBn4C6BzWt2F4A9ftdnKFtOtGprSVjttlqtJVQZ

(Press/Click on the above link to be automatically sent to the web page.)

Please make the correction(s) as specified above and resubmit your paper following the same steps as before.

If you have any questions please feel free to contact us.

Sincerely,

Editorial Office
Cell Death & Disease
cddisease@springernature.com

Subject:	Re: Change of Attribution Agreement
From:	"王雅梅" <ymwang@ccmu.edu.cn> Feb 22, 2022 7:23:45 PM
To:	"李纤" <qianli@ccmu.edu.cn>
Cc:	15810756234@163.com, wuweihua@mail.ccmu.edu.cn, jingliu8762@163.com, tinglanzs@163.com, xzn1016@126.com, gaikaiyuan2000@163.com, huliye@mail.ccmu.edu.cn, zhaoliluo@mail.ccmu.edu.cn, chaowei@ccmu.edu.cn, wangxt1414@163.com, luyabin@ccmu.edu.cn, chzhang@ccmu.edu.cn, wangpp@ccmu.edu.cn, zuozt@ibp.ac.cn, feiyang@ccmu.edu.cn

Dear Professor Li,

I agree to add Dr. Jing Liu and Ms. Xiaotong Wang as coauthors to this manuscript entitled "Ferroptosis in oligodendrocyte progenitor cells mediates white matter injury after hemorrhagic stroke"

Best wishes,

Yamei Wang

> -----原始邮件-----
> 发件人:"李纤" <qianli@ccmu.edu.cn>
> 发送时间:2022-02-22 18:25:15 (星期二)
> 收件人: 15810756234@163.com, wuweihua@mail.ccmu.edu.cn, jingliu8762@163.com, tinglanzs@163.com, xzn1016@126.com, gaikaiyuan2000@163.com, huliye@mail.ccmu.edu.cn, zhaoliluo@mail.ccmu.edu.cn, chaowei@ccmu.edu.cn, wangxt1414@163.com, luyabin@ccmu.edu.cn, ymwang@ccmu.edu.cn, chzhang@ccmu.edu.cn, wangpp@ccmu.edu.cn, zuozt@ibp.ac.cn, feiyang@ccmu.edu.cn
> 抄送: qianli@ccmu.edu.cn
> 主题: Change of Attribution Agreement
>
> Dear co-authors,
>
> As you know, we have added Dr. Jing Liu and Ms. Xiaotong Wang as coauthors to this manuscript entitled "Ferroptosis in oligodendrocyte progenitor cells mediates white matter injury after hemorrhagic stroke". Please reply to my email and confirm that you have agreed with these changes.
>
> Best wishes,
>
> Qian Li
>
> ----------------
>
> Dear Professor Li,
>
> In checking in your manuscript submitted to Cell Death & Disease it has come to our attention that the following must be addressed before we can proceed.
>
> 1. It has come to our attention that your most recent author list differs from the one in your original submission.
>
> Please request agreement from all authors including additions and deletions, these can be collected in the following way:

Email your co-authors with the change, and ask them to reply to your email confirming that they agree to these changes. Once you have collected these replies, please combine all of the co-authors' email responses in one document and upload this file to your submission.

Your paper is now available for you to edit, you may access via the following link:

https://mts-cddis.nature.com/cgi-bin/main.plex?el=A7CA3WBn4C6BzWt2F4A9ftdnKFtOtGprSVjttlqtJVQZ

(Press/Click on the above link to be automatically sent to the web page.)

Please make the correction(s) as specified above and resubmit your paper following the same steps as before.

If you have any questions please feel free to contact us.

Sincerely,

Editorial Office
Cell Death & Disease
cddisease@springernature.com

Subject:	Re: Change of Attribution Agreement
From:	"张晨光" <chzhang@ccmu.edu.cn> Feb 22, 2022 7:18:05 PM
To:	"李纤" <qianli@ccmu.edu.cn>

Dear Prof. Li,

I agreed to add Dr.Jing Liu and MS.Xiaotong Wang as coauthors due to their contributions to this work.

Best Wishes!

Chenguang Zhang

-----原始邮件-----
发件人:"李纤" <qianli@ccmu.edu.cn>
发送时间:2022-02-22 18:25:15 (星期二)
收件人: 15810756234@163.com, wuweihua@mail.ccmu.edu.cn, jingliu8762@163.com, tinglanzs@163.com, xzn1016@126.com, gaikaiyuan2000@163.com, huliye@mail.ccmu.edu.cn, zhaoliluo@mail.ccmu.edu.cn, chaowei@ccmu.edu.cn, wangxt1414@163.com, luyabin@ccmu.edu.cn, ymwang@ccmu.edu.cn, chzhang@ccmu.edu.cn, wangpp@ccmu.edu.cn, zuozt@ibp.ac.cn, feiyang@ccmu.edu.cn
抄送: qianli@ccmu.edu.cn
主题: Change of Attribution Agreement

Dear co-authors,

As you know, we have added Dr. Jing Liu and Ms. Xiaotong Wang as coauthors to this manuscript entitled "Ferroptosis in oligodendrocyte progenitor cells mediates white matter injury after hemorrhagic stroke". Please reply to my email and confirm that you have agreed with these changes.

Best wishes,

Qian Li

Dear Professor Li,

In checking in your manuscript submitted to Cell Death & Disease it has come to our attention that the following must be addressed before we can proceed.

1. It has come to our attention that your most recent author list differs from the one in your original submission.

Please request agreement from all authors including additions and deletions, these can be collected in the following way:

Email your co-authors with the change, and ask them to reply to your email confirming that they agree to these changes. Once you have collected these replies, please combine all of the co-authors' email responses in one document and upload this file to your submission.

Your paper is now available for you to edit, you may access via the following link:

https://mts-cddis.nature.com/cgi-bin/main.plex?el=A7CA3WBn4C6BzWt2F4A9ftdnKFtOtGprSVjttlqtJVQZ

(Press/Click on the above link to be automatically sent to the web page.)

Please make the correction(s) as specified above and resubmit your paper following the same steps as before.

If you have any questions please feel free to contact us.

Sincerely,

Editorial Office
Cell Death & Disease
cddisease@springernature.com

张晨光

Chenguang Zhang, PhD

Department of Biochemistry and Molecular biology

Capital Medical University

Beijing, China

Tel: (86)010-83950517/1

Subject:	Re: Change of Attribution Agreement
From:	"王培培" <wangpp@ccmu.edu.cn> Feb 22, 2022 7:50:22 PM
To:	"李纤" <qianli@ccmu.edu.cn>

Dear Dr. Li,

I agree with the authorship change.
Thanks.

Best,
Peipei Wang

-----Original Messages-----
From:"李纤" <qianli@ccmu.edu.cn>
Sent Time:2022-02-22 18:25:15 (Tuesday)
To: 15810756234@163.com, wuweihua@mail.ccmu.edu.cn, jingliu8762@163.com, tinglanzs@163.com, xzn1016@126.com, gaikaiyuan2000@163.com, huliye@mail.ccmu.edu.cn, zhaoliluo@mail.ccmu.edu.cn, chaowei@ccmu.edu.cn, wangxt1414@163.com, luyabin@ccmu.edu.cn, ymwang@ccmu.edu.cn, chzhang@ccmu.edu.cn, wangpp@ccmu.edu.cn, zuozt@ibp.ac.cn, feiyang@ccmu.edu.cn
Cc: qianli@ccmu.edu.cn
Subject: Change of Attribution Agreement

Dear co-authors,

As you know, we have added Dr. Jing Liu and Ms. Xiaotong Wang as coauthors to this manuscript entitled "Ferroptosis in oligodendrocyte progenitor cells mediates white matter injury after hemorrhagic stroke". Please reply to my email and confirm that you have agreed with these changes.

Best wishes,

Qian Li

Dear Professor Li,

In checking in your manuscript submitted to Cell Death & Disease it has come to our attention that the following must be addressed before we can proceed.

1. It has come to our attention that your most recent author list differs from the one in your original submission.

Please request agreement from all authors including additions and deletions, these can be collected in the following way:

Email your co-authors with the change, and ask them to reply to your email confirming that they agree to these changes. Once you have collected these replies, please combine all of the co-authors' email responses in one document and upload this file to your submission.

Your paper is now available for you to edit, you may access via the following link:

https://mts-cddis.nature.com/cgi-bin/main.plex?el=A7CA3WBn4C6BzWt2F4A9ftdnKFtOtGprSVjttlqtJVQZ

(Press/Click on the above link to be automatically sent to the web page.)

Please make the correction(s) as specified above and resubmit your paper following the same steps as before.

If you have any questions please feel free to contact us.

Sincerely,

Editorial Office
Cell Death & Disease
cddisease@springernature.com

Subject:	Re: Change of Attribution Agreement
From:	"zhentao zuo" <zuozt@ibp.ac.cn> Feb 22, 2022 6:57:39 PM
To:	"李纤" <qianli@ccmu.edu.cn>
Cc:	15810756234@163.com, wuweihua@mail.ccmu.edu.cn, jingliu8762@163.com, tinglanzs@163.com, xzn1016@126.com, gaikaiyuan2000@163.com, huliye@mail.ccmu.edu.cn, zhaoliluo@mail.ccmu.edu.cn, chaowei@ccmu.edu.cn, wangxt1414@163.com, luyabin@ccmu.edu.cn, ymwang@ccmu.edu.cn, chzhang@ccmu.edu.cn, wangpp@ccmu.edu.cn, feiyang@ccmu.edu.cn

Dear Prof. Li,
Congratulations. I agreed to add them as coauthor due to their contributions to this work.
Bests!

Zhentao Zuo

On Feb 22, 2022, at 6:25 PM, 李纤 <qianli@ccmu.edu.cn> wrote:

Dear co-authors,

As you know, we have added Dr. Jing Liu and Ms. Xiaotong Wang as coauthors to this manuscript entitled "Ferroptosis in oligodendrocyte progenitor cells mediates white matter injury after hemorrhagic stroke". Please reply to my email and confirm that you have agreed with these changes.

Best wishes,

Qian Li

Dear Professor Li,

In checking in your manuscript submitted to Cell Death & Disease it has come to our attention that the following must be addressed before we can proceed.

1. It has come to our attention that your most recent author list differs from the one in your original submission.

Please request agreement from all authors including additions and deletions, these can be collected in the following way:

Email your co-authors with the change, and ask them to reply to your email confirming that they agree to these changes. Once you have collected these replies, please combine all of the co-authors' email responses in one document and upload this file to your submission.

Your paper is now available for you to edit, you may access via the following link:

https://mts-cddis.nature.com/cgi-bin/main.plex?el=A7CA3WBn4C6BzWt2F4A9ftdnKFtOtGprSVjttlqtJVQZ

(Press/Click on the above link to be automatically sent to the web page.)

Please make the correction(s) as specified above and resubmit your paper following the same steps as before.

If you have any questions please feel free to contact us.

Sincerely,

Editorial Office
Cell Death & Disease

cddisease@springernature.c
om

Subject:	Re: Change of Attribution Agreement
From:	"杨菲" <feiyang@ccmu.edu.cn> Feb 22, 2022 7:05:21 PM
To:	"李纤" <qianli@ccmu.edu.cn>

Hi Dr. Li,

I agree with the changes.

Fei Yang

-----原始邮件-----
发件人:"李纤" <qianli@ccmu.edu.cn>
发送时间:2022-02-22 18:25:15 (星期二)
收件人: 15810756234@163.com, wuweihua@mail.ccmu.edu.cn, jingliu8762@163.com, tinglanzs@163.com, xzn1016@126.com, gaikaiyuan2000@163.com, huliye@mail.ccmu.edu.cn, zhaoliluo@mail.ccmu.edu.cn, chaowei@ccmu.edu.cn, wangxt1414@163.com, luyabin@ccmu.edu.cn, ymwang@ccmu.edu.cn, chzhang@ccmu.edu.cn, wangpp@ccmu.edu.cn, zuozt@ibp.ac.cn, feiyang@ccmu.edu.cn
抄送: qianli@ccmu.edu.cn
主题: Change of Attribution Agreement

Dear co-authors,

As you know, we have added Dr. Jing Liu and Ms. Xiaotong Wang as coauthors to this manuscript entitled "Ferroptosis in oligodendrocyte progenitor cells mediates white matter injury after hemorrhagic stroke". Please reply to my email and confirm that you have agreed with these changes.

Best wishes,

Qian Li

Dear Professor Li,

In checking in your manuscript submitted to Cell Death & Disease it has come to our attention that the following must be addressed before we can proceed.

1. It has come to our attention that your most recent author list differs from the one in your original submission.

Please request agreement from all authors including additions and deletions, these can be collected in the following way:

Email your co-authors with the change, and ask them to reply to your email confirming that they agree to these changes. Once you have collected these replies, please combine all of the co-authors' email responses in one document and upload this file to your submission.

Your paper is now available for you to edit, you may access via the following link:

https://mts-cddis.nature.com/cgi-bin/main.plex?el=A7CA3WBn4C6BzWt2F4A9ftdnKFtOtGprSVjttlqtJVQZ

(Press/Click on the above link to be automatically sent to the web page.)

Please make the correction(s) as specified above and resubmit your paper following the same steps as before.

If you have any questions please feel free to contact us.

Sincerely,

Editorial Office
Cell Death & Disease
cddisease@springernature.com
